# Compositional Gradient–Engineered Ti–WO_3_ Films for Simultaneous Enhancement of Coloration Efficiency and Mechanical Robustness

**DOI:** 10.1002/smll.202514826

**Published:** 2026-03-23

**Authors:** Fang Luo, Chang‐Shin Park, Yeoung‐Eun Seo, Xiaosong Jiang, Han‐Ki Kim

**Affiliations:** ^1^ School of Advanced Materials Science and Engineering Sungkyunkwan University Suwon Gyunggi‐do Republic of Korea; ^2^ School of Materials Science and Engineering Southwest Jiaotong University Chengdu Sichuan China; ^3^ Department of Display Engineering Sungkyunkwan University Suwon Gyunggi‐do Republic of Korea

**Keywords:** co‐sputtering, electro‐optical performance, gradient Ti doping, oxygen vacancy modulation, WO_3_ electrochromic films

## Abstract

Electrochromic devices offer immense potential for energy‐saving and adaptive optics, yet their advancement is hindered by slow ion diffusion and low charge utilization, which critically limit the development of next‐generation flexible optoelectronic technologies. In this study, a gradient‐engineered Ti‐doped WO_3_ architecture is developed to enable robust electron–ion coupling, leading to enhanced coloration efficiency and mechanical robustness. By dynamically modulating the sputtering powers of TiO_2_ and W metal targets, a continuous Ti concentration gradient was established, forming a self‐built internal electric field that promotes electron–ion synergy and accelerates Li^+^ transport. The optimized gradient film delivers a large optical modulation of 78.9% and a high coloration efficiency (CE) of 137.4 cm^2^ C^−1^, outperforming uniformly doped counterparts. The gradient structure suppresses abrupt band offsets and induces smooth energy band bending across the film, facilitating fast redox kinetics and enhanced reversibility. Furthermore, after 500 bending cycles, the film retains over 82% of its modulation amplitude and exhibits an increased CE of 213.73 cm^2^ C^−1^, confirming outstanding flexibility and stress adaptability. This gradient doping strategy unites the structural continuity of homojunctions with band engineering of heterojunctions, offering a universal design paradigm for high‐performance flexible electrochromic and photoelectronic systems.

## Graphical abstract

A gradient Ti‐doped WO_3_ electrochromic film is developed via dynamic co‐sputtering, enabling depth‐dependent band engineering and strain regulation. The gradient structure induces a built‐in electric field across the film thickness that enhances coupled ion–electron transport while improving mechanical robustness. This work establishes a general gradient‐driven strategy for high‐performance, flexible electrochromic materials and devices.

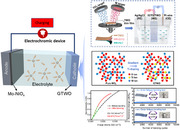

## 1 Introduction

Electrochromic (EC) materials, capable of reversible modulation of optical properties under a low voltage, have emerged as key components for energy‐efficient windows, rearview mirrors, information displays, and smart wearable systems [1, 2, 3, 4, 5, 6]. Their unique ability to reversibly switch between transparent and colored states through ion–electron intercalation enables dynamic control over light and heat transmission, offering a pathway toward sustainable and responsive optoelectronic architectures. A wide variety of materials exhibit EC properties, including inorganic metal oxides, organic conjugated polymers, and organic–inorganic hybrid systems [4, 7, 8, 9]. Among these candidates, tungsten trioxide (WO_3_), as an inorganic transition metal oxide, has become the most promising cathodic electrochromic material due to its excellent optical modulation ability, fast response time, outstanding cycling stability, low cost, and ease of fabrication [9, 10, 11, 12, 13]. Previous studies have demonstrated that the morphology of WO_3_ films and the configuration of electrodes play a key role in influencing electrolyte interface chemistry and ion diffusion processes in EC devices [14, 15, 16, 17, 18]. For example, amorphous WO_3_ (a‐WO_3_) exhibits high coloration efficiency (CE) and rapid response because of the disordered arrangement of [WO_6_] octahedra, but it suffers from poor stability [1, 19, 20, 21]. In contrast, crystalline WO_3_ (c‐WO_3_) offers superior durability due to its ordered structure and low dissolution rate; however, its relatively dense structure can hinder ion diffusion and slow the response speed [20, 22, 23]. Lopa et al. [15]. prepared wafer‐scale nanocomposite electrodes of WO_3_‐MoO_3_ with heterostructures via atomic layer deposition, demonstrating high purity, well‐defined crystallinity, and uniform morphology, enabling efficient pseudocapacitive Faradaic redox reactions with good reversibility. However, the fabrication of such heterostructures is complex, and their interfacial stability still requires further optimization. Therefore, how to improve the EC performance of amorphous WO_3_ films while overcoming the limitations of crystalline and heterostructured films remains a critical research direction.

To simplify fabrication processes and enhance the performance of WO_3_‐based EC films and devices, various quality optimization strategies have been explored, including doping [13], porous structures [24], and core–shell architectures [1], etc. In particular, metal doping has emerged as an effective approach to tailor the local electronic structure and modulate ion diffusion pathways in WO_3_ films [25, 26]. Dopants such as Ti, Mo, Ag, and Ni can introduce controlled lattice distortions and oxygen vacancies, facilitating charge transfer and mitigating Li^+^ trapping [4, 27, 28]. In common lithium‐ion electrolyte environments, WO_3_ films experience performance degradation during electrochemical cycling, mainly due to the continuous accumulation of lithium ions at W═O bonds, W─O─H bonds, and other trapping sites in the film, leading to the formation of lithium tungstate and thus affecting electrochemical stability [27, 29]. Among various dopants, Ti doping has shown exceptional potential: it suppresses the formation of irreversible Li─W─O bonds, stabilizes the amorphous network, and enhances both ion insertion kinetics and electrochemical reversibility [19, 30]. Yet, the improvement is highly sensitive to dopant distribution and concentration [17, 31]. For instance, Han et al. [19]. found that increasing nanocrystalline TiO_2_ content to 30 wt.% in amorphous WO_3_ sped up charge deintercalation sevenfold but reduced bleaching transmittance. Chen et al. [32]. showed that Ti‐doped WO_3_ films made by reactive sputtering under optimal oxygen flow achieved high optical modulation (71.7% at 600 nm), fast switching, and excellent CE. Therefore, further optimization of ion transport and stability in Ti‐doped WO_3_ films while maintaining high transmittance is a pressing research challenge. The long‐term stability of ECD depends not only on the electrochemical performance of the active layer but also critically on the adhesion quality between the EC layer and the substrate. Studies have reported that insufficient interfacial adhesion can cause film delamination, crack propagation, and optical performance degradation during cycling, especially pronounced in flexible substrates and thick films [12, 33, 34, 35, 36, 37]. To enhance interface stability, various strategies have been attempted, including introducing buffer layers such as TiO_2_ or ITO to optimize chemical bonding at the interface [12, 38] and controlling film microstructures (e.g., nanocolumns, nanopores) to improve mechanical interlocking [39]. However, these approaches often require additional interface layers or complex templating processes, increasing fabrication cost and complexity. Furthermore, WO_3_ films prepared by purely chemical methods typically exhibit novel nanostructures but suffer from poor adhesion to substrates [40, 41]. In contrast, WO_3_ films fabricated by pure physical methods such as vacuum thermal evaporation and magnetron sputtering offer advantages including high film density, precise control over composition and thickness, high purity, and strong adhesion to various substrates [26, 33]. Nevertheless, their dense structure may hinder rapid ion migration, thus affecting EC response speed and CE [10, 33].

In this work, a gradient‐doping strategy is proposed for Ti–WO_3_ (TWO) films by employing dynamic co‐sputtering of TiO_2_ and W metal targets. By continuously modulating the Ti concentration along the film thickness, a band‐graded architecture is constructed that differs fundamentally from conventional uniformly doped WO_3_ systems. Unlike previously reported Ti‐doped or composite WO_3_ electrochromic films that focus primarily on compositional optimization, this study introduces a depth‐resolved gradient structure to regulate electron and ion transport in a synergistic manner. The gradient design is expected to induce a built‐in potential across the film depth, which facilitates coupled electron–ion migration while alleviating interfacial strain and mechanical mismatch. Through this approach, the present work bridges the conceptual advantages of homojunction continuity and heterojunction band engineering, offering a new pathway to optimize electrochromic materials via gradient‐driven structure–property relationships. The primary objective of this study is to elucidate the role of gradient Ti incorporation in modulating the electrochemical, optical, and mechanical behaviors of WO_3_‐based electrochromic films, thereby providing a general design strategy for high‐performance and mechanically robust electrochromic systems.

## 2 Results and Discussion

**FIGURE 1:**
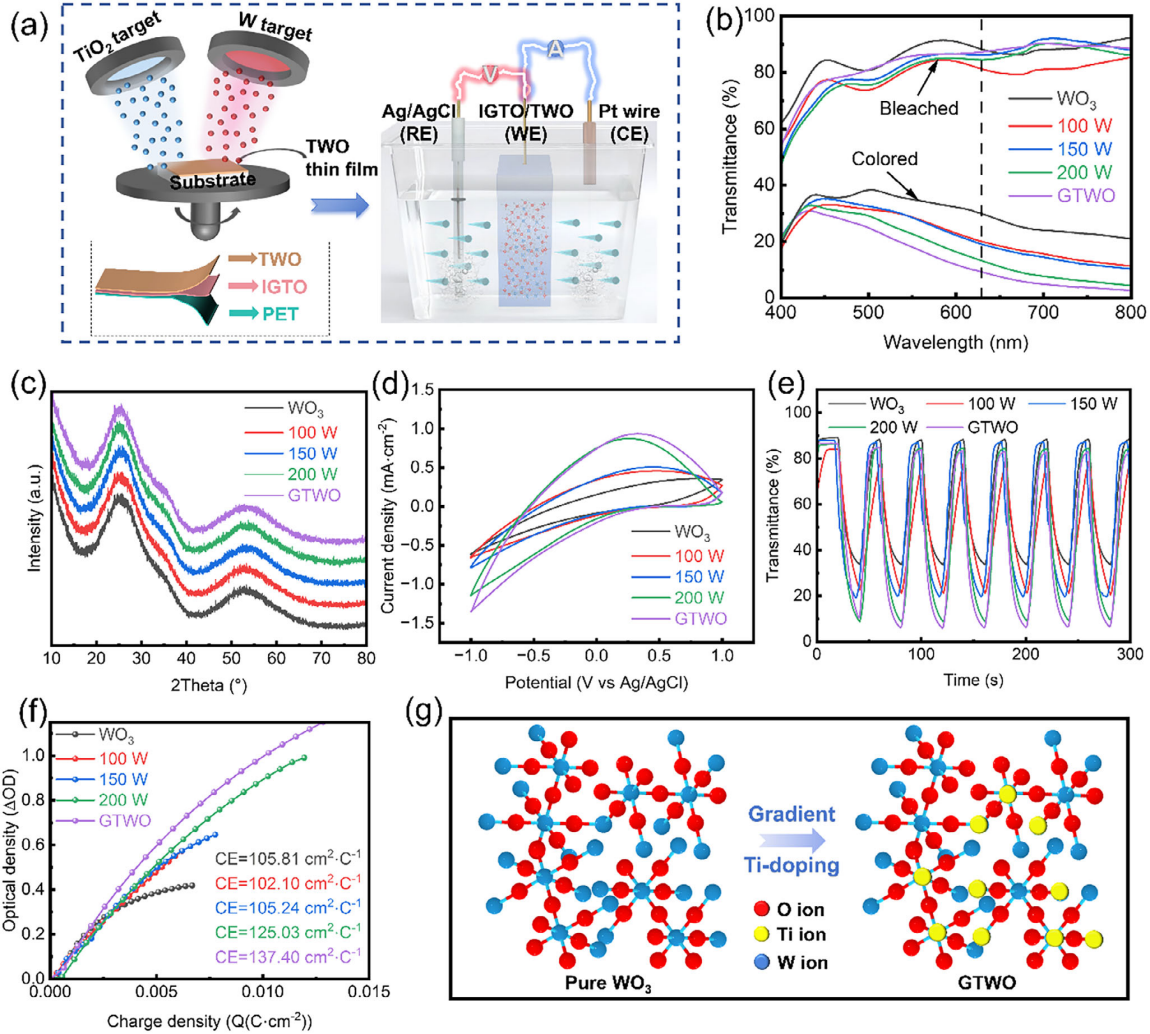
(a) Schematic illustration of the RF/DC co‐sputtering system for depositing TWO/IGTO/PET and the electrochemical test configuration. Performance of TWO films deposited at different sputtering powers of TiO_2_ target and gradient doping: (b) Optical transmittance spectra in the colored state and the bleached state, (c) XRD patterns, (d) cyclic voltammetry (CV) curves, (e) transmittance modulation during repeated coloration/bleaching cycles and (f) optical density vs. charge density plots used to calculate coloration efficiency. (g) Structural models of pure WO_3_ and GTWO films.

The TWO films were fabricated via a dual‐target magnetron co‐sputtering process, where the RF and DC sputtering powers applied to TiO_2_ and W targets, respectively, were dynamically adjusted to control Ti incorporation. This configuration enables precise modulation of the Ti gradient along the film thickness, yielding both uniform and gradient‐doped structures as shown in Figure 1a. Figure S1 displays an actual photograph of the co‐sputtering process. The optimized gradient film combines the electronic benefits of Ti doping with the structural coherence of amorphous WO_3_.

The structure and sputtering power schematic are shown in Figure S2. The film thickness (330 nm) was chosen based on the optimized parameters reported by Park et al. [34]. to ensure a balance between optical contrast and switching speed. As discussed in recent surface‐engineering studies [42], film thickness significantly influences the ion diffusion path and interfacial charge‐transfer resistance. In the study, this thickness allows for sufficient active sites for ion intercalation while maintaining a relatively short diffusion length, thereby optimizing the coloration efficiency. Concurrently, GTWO films were fabricated by dynamically regulating the sputtering power of both targets. As shown in Figure S3, all as‐sputtered films exhibited high transmittance across the visible to near‐infrared spectrum, with average visible transmittance (AVT) (400–800 nm) maintained between 69.88% and 72.27%. Compared to pure WO_3_, the AVT of TWO films is essentially equivalent (with a difference maintained within 2%), indicating that Ti doping and gradient control do not significantly reduce the optical transparency of the films. To evaluate the optical modulation capability of the materials, the spectral response characteristics of various samples in the visible‐to‐near‐infrared region were compared. Figure 1b indicates that all samples maintained high transparency in the bleached state. Upon transitioning to the colored state, the transmittance of each sample decreased significantly, exhibiting distinct electrochromic behavior. A comparison reveals that the pure WO_3_ thin film has limited modulation depth in the long‐wavelength region. In contrast, the GTWO sample achieved lower transmittance in the colored state, demonstrating superior optical contrast. This suggests that process optimization or compositional adjustment effectively enhanced the material's light absorption and modulation capabilities. All pure WO_3_ and TWO films prepared at room temperature by RF sputtering in this study exhibited an amorphous structure, as confirmed by the XRD pattern shown in Figure 1c. The amorphous WO_3_ structure is favorable for electrochromic performance due to its open network of [WO_6_] octahedra, which provides accessible ion diffusion channels and mitigates structural strain during repeated Li^+^ insertion/extraction cycles. In highly crystalline structures, ions are tightly packed, hindering material diffusion and resulting in slower response times. Amorphous WO_3_ coatings, characterized by their loosely packed crystal structure and sufficient specific surface area, demonstrate fast switching kinetics and substantial optical modulation capabilities [43]. The electrochemical behavior of pure WO_3_, uniformly doped TWO, and GTWO films was analyzed using an electrochemical workstation. This facilitates the investigation of electrochemical characteristics during lithiation (charge insertion) and delithiation (charge deintercalation) processes, thereby providing deeper insights into the formation mechanism of optical tunability [44]. Figure 1d shows the insertion and extraction processes of Li^+^ in different films during cycling in the range from −1 to 1 V at a scan rate of 100 mV s^−1^. The CV curves all exhibit characteristic duckbill profiles, consistent with those reported in previous literature [1, 24, 34, 44, 45, 46, 47]. All films exhibit identifiable and broad oxidation peaks consistent with the conventional behavior of amorphous WO_3_^1^. The CV curves show that the reduction current density increases steadily with higher Ti doping, indicating a significantly enhanced current response. This enhancement mainly arises from improved electronic conductivity, lower charge‐transfer resistance, favorable band‐structure modification, and more active sites introduced by Ti, all of which accelerate the reduction reaction kinetics [30, 48].

Compared to pure WO_3_, TWO films exhibit faster ion insertion/extraction kinetics. Notably, the GTWO film exhibits the highest peak current density, indicating that the gradient structure possesses higher electrochemical activity, with doping enhancing charge storage capacity. As shown in Figure 1e, the transmittance of different films was measured at a fixed wavelength of 630 nm. Electrochemical energy storage performance was evaluated by measuring the in situ visible transmittance changes of different films over 5 min under applied operating potentials of ±1 V. According to the Faughnan model [49], the color change in WO_3_ thin films, acting as cathodic electrochromic materials, is caused by the dual insertion/deinsertion redox reaction of Li^+^ and electrons. The switching process between the colored and bleached states can be attributed to the following electrochemical reaction [3, 34, 50, 51, 52]:

(1) $$WO_{3}\;\left(bleached\right)+xLi^{+}+xe^{+}↔Li_{x}WO_{3}\left(colored\right)$$

This phenomenon is also corroborated by the increase in optical density (ΔOD) within the gradient film. Optical density is used to calculate the film's CE, a parameter measuring the quality of electrochromic performance. CE is defined as the change in optical contrast corresponding to the amount of charge injected per unit area of electrode [53]. The calculation formula is as follows (Equation 2) [34, 53, 54, 55, 56]:

(2) $$CE=\Delta OD/\left(\frac{Q_{i}}{A}\right)$$

(3) $$\Delta OD=\log\left(t_{b}/t_{c}\right)$$

Here, Δ*OD* denotes the optical density calculated according to Equation (3), *t_b_* and *t_c_* represent the optical transmittance of the film in the bleached and colored states, respectively, and $\frac{Q_{i}}{A}$ defines the charge density during the electrochemical process. GTWO film achieved a CE of ∼137.4 cm^2^ C^−1^, representing a significant improvement over the pure WO_3_ film (∼105.81 cm^2^ C^−1^), as shown in Figure 1f. As shown in Figure S4, the CE of different samples was quantitatively extracted from the slope of ΔOD versus charge density by linear fitting within the same low‐charge‐density region, revealing a systematic enhancement in CE upon Ti doping and gradient structure design. Compared to pure WO_3_ film, the gradient film with higher CE achieved greater optical modulation while inserting or removing the same number of ions.

Figure 1g compares the structural mechanism of WO_3_ and GTWO films. In pure WO_3_ amorphous film, ion diffusion pathways are limited due to the locally disordered network structure. Gradient Ti doping introduces compositional inhomogeneity and oxygen vacancies into the film, which not only extends ion migration paths but also helps alleviate the accumulation of local stress during repeated cycles. Thus, the superior electrochromic performance of GTWO film primarily stems from the synergistic effects of improved ion diffusion kinetics and enhanced structural stability.

**FIGURE 2:**
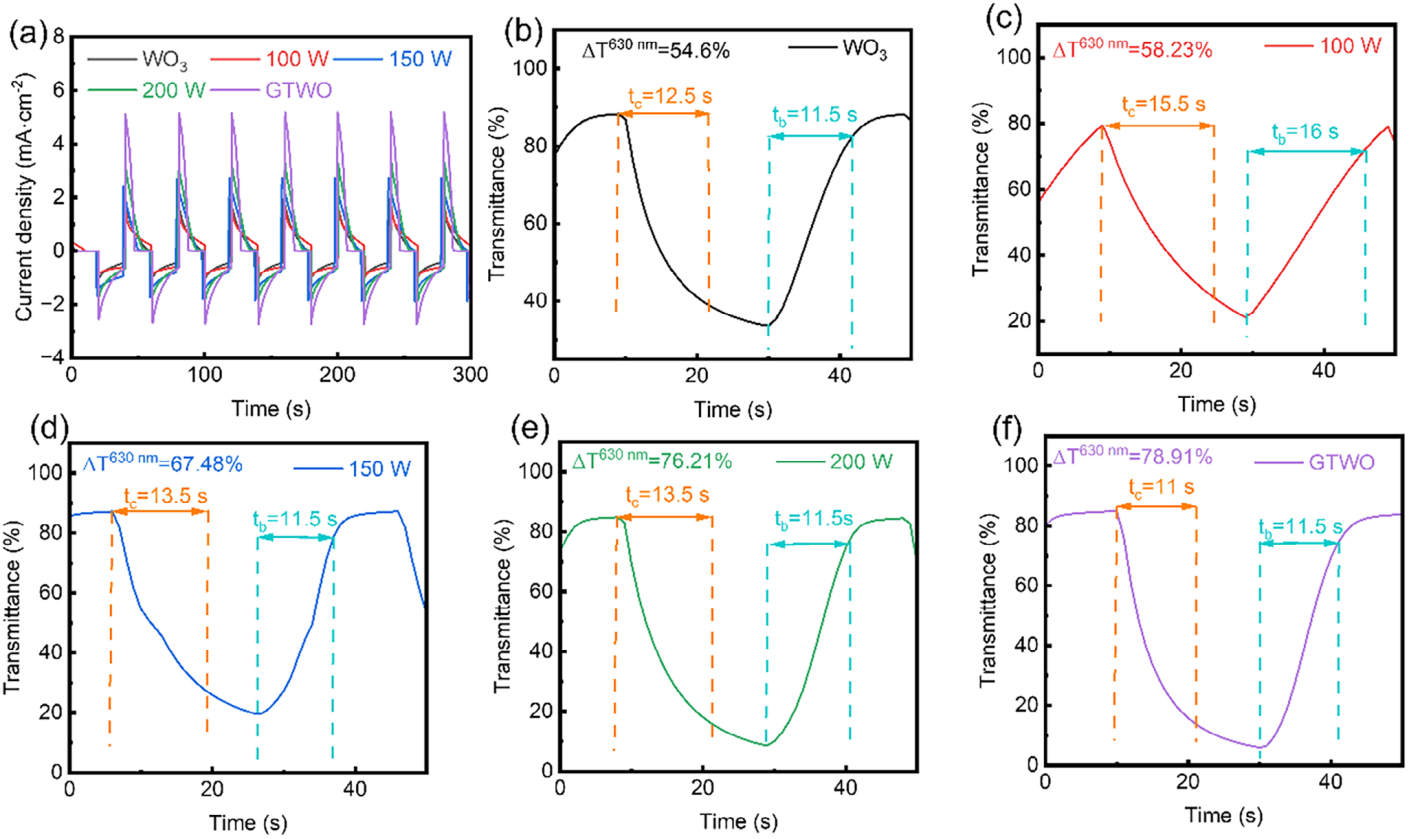
Electrochromic performance of TWO/IGTO/PET structures deposited under different sputtering powers of TiO_2_ and gradient films. (a) Chronoamperometry curves during repeated coloring/bleaching cycles. Response times for (b) pure WO_3_, (c) 100 W TWO, (d) 150 W TWO, (e) 200 W TWO, and (f) GTWO films, showing the corresponding color modulation (ΔT), coloring time (t_c_), and bleaching time (t_b_) were observed at 630 nm.

Further validation of the GTWO film's faster and more stable current response under potential switching was demonstrated in the chronoamperometric test, as shown in Figure 2a. Simultaneously, all films exhibited stable and reversible current responses throughout multiple dyeing/bleaching cycles, indicating excellent reversibility in their electrochromic process. Response time—the duration required to transition between electrochromic states—is a critical parameter for evaluating the suitability of electrochromic devices for various applications [55, 57]. Transmittance modulation curves at 630 nm wavelength in Figure 2b–f further reveal changes in electrochromic performance. Table 1 shows the time required for pure WO_3_, uniformly doped TWO, and GTWO films to achieve 90% of the total optical change under square‐wave voltage pulses. The bleaching times for the five different films were 12.5, 15.5, 13.5, 13.5, and 11 s, respectively, with corresponding coloring times of 11.5, 16, 11.5, 11.5, and 11.5 s. The incorporation of Ti into WO_3_ enhances performance by providing superior optical modulation capability, resulting in more pronounced transparency changes and more efficient switching. This performance improvement stems from two films’ superior ion storage capacity and enhanced ionic conductivity, which reduces internal resistance and facilitates more efficient electrochromic charge transport within the device. Notably, as shown in Figure 2f, the GTWO EC film exhibits shorter bleaching and recoloring durations, along with the highest optical modulation amplitude (ΔT = 78.9%). Compared to the EC layer of GTWO, the optical modulation performance of the undoped and uniformly doped EC layers is significantly inferior, resulting in prolonged switching times. The Video S1 demonstrates the entire process of coloring and bleaching of GTWO.

**TABLE 1:** Electrochromic parameters of TWO/IGTO/PET structures deposited under different sputtering powers of TiO_2_ and gradient films, including bleached transmittance, colored transmittance, ΔT, t_c_, t_b_, and CE.

Samples	Bleached Transmittance [%]	Colored Transmittance [%]	Color Modulation [%]	Bleached Time [s]	Colored Time [s]	Coloration Efficiency [cm^2^ C^−1^]
WO_3_	88.26	33.66	54.6	12.5	11.5	105.81
0 W	79.45	21.22	58.23	15.5	16	102.10
150 W	87.17	19.69	67.48	13.5	11.5	105.24
200 W	84.87	8.66	76.21	13.5	11.5	125.03
GTWO	84.91	6.00	78.91	11	11.5	137.40

This finding demonstrates that the gradient doping structure provides more active sites at the GTWO film surface to facilitate rapid ion insertion while maintaining an efficient transport channel within the film. This enables the achievement of excellent kinetic performance alongside high contrast. This pattern aligns with research findings indicating that gradient‐doped structures can effectively enhance carrier separation and transport.

For instance, Srivastav et al. [58]. significantly improved photoresponse by constructing a gradient structure through Ti doping in Fe_2_O_3_, while Luo et al. [59]. enhanced charge separation efficiency in Fe_2_O_3_ nanoarrays via P‐gradient doping, while recently reported oxygen gradient doping also boosted photoelectrochemical activity by introducing an internal electric field [60]. Collectively, these findings demonstrate that gradient doping structures can simultaneously optimize both interfacial and bulk transport properties, thereby delivering outstanding comprehensive performance in electrochromic systems.

**FIGURE 3:**
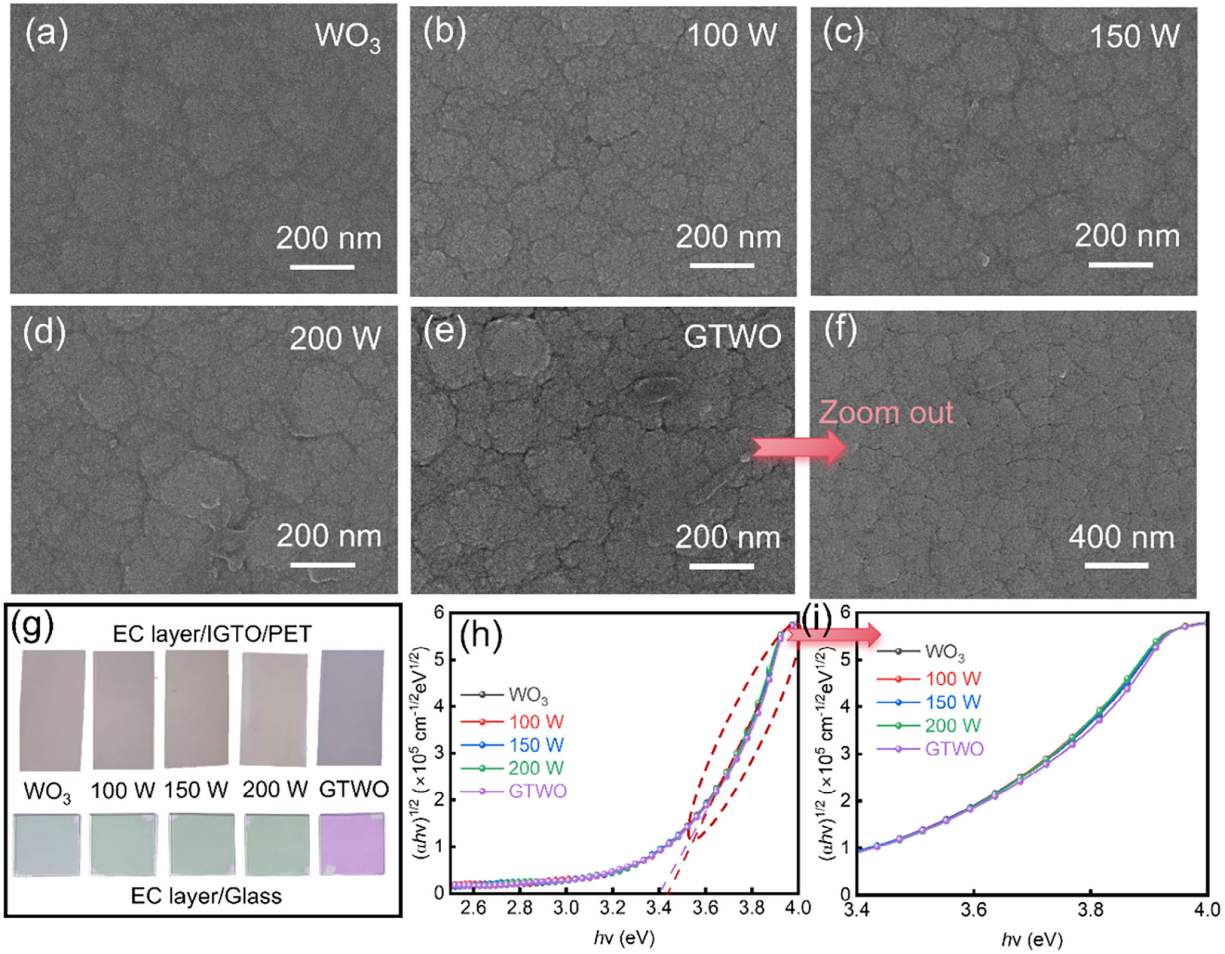
High‐magnification SEM images of films deposited under different sputtering powers of TiO_2_ and gradient doping: (a) pure WO_3_, (b) 100 W TWO, (c) 150 W TWO, (d) 200 W TWO, (e) GTWO films. (f) Low‐magnification SEM images of GTWO films. (g) Actual pictures of different films on IGTO/PET and glass substrates. (h) Calculated Tauc plots. (i) Tauc plots from 3.4 to 4.0 eV.

The microstructure of thin films directly influences their optical and electrochemical properties. The high‐magnification field emission scanning electron microscope (FESEM) images in Figure 3a–f reveal that all thin films exhibit a smooth, uniform surface morphology characterized by a lack of distinct grain boundaries. This morphology is consistent with amorphous or nanocrystalline film structures, which aligns well with the preceding XRD results. In particular, the GTWO film (Figure 3f), with its fine, nanostructured features, further suggests a quasi‐amorphous structure possessing high interface and/or defect densities, which is beneficial for the intercalation and de‐intercalation of ions [61]. The smooth and uniform morphology ensures a continuous and accessible surface, which facilitates electrolyte penetration and improves interfacial contact. This shortens the diffusion distance of conductive ions and enhances GTWO‐based electrochromic performance [9]. Figure 3g shows the actual pictures of different films on IGTO/PET and glass substrates. It can be observed that films prepared under different conditions exhibit completely different colors on the same substrate, indicating that even minimal Ti doping significantly affects the film color. The distinct colors in the actual film pictures indirectly confirm the successful Ti doping. It is also evident that further Ti doping is undesirable, as it would further reduce film transparency and consequently impair electrochromic performance. The gradient‐doped films exhibit markedly different colors compared to uniformly doped films, primarily due to local variations in bandgap, refractive index, and grain structure caused by the Ti concentration gradient. These variations result in distinct light absorption and interference effects across different regions. The optical bandgap was estimated using Tauc plots extracted from the UV–vis absorption spectra. The Tauc plots for corresponding films are shown in Figure 3h, i. Considering indirect transition effects, the bandgap was calculated using the following Equation [38, 62]:

(4) $${\left(\alpha hv\right)}^{1/2}=A\left(hv-E_{g}\right)$$

Here, α is the absorption coefficient, *hv* is the photon energy (eV), A is the proportional constant, and *E_g_* is the optical bandgap (eV). Although pristine WO_3_ is generally regarded as an indirect bandgap semiconductor, Ti incorporation introduces local structural distortion and defect‐related states, which weaken the phonon‐assisted transition requirement. As a result, Ti‐doped WO_3_ exhibits quasi‐direct optical transition characteristics in UV–vis absorption analysis, while retaining an indirect bandgap nature from an electronic structure perspective [38]. It can be observed that compared to pure WO_3_ films, the bandgap change is negligible as the TiO_2_ sputtering power increases to 200 W. However, the bandgap of the GTWO film is slightly lower than that of the TWO films. This phenomenon is clearly observable in the bandgap magnification shown in Figure 3i. This bandgap difference indicates that the gradient interface may also exhibit band alignment characteristics similar to quasi‐heterojunctions, thereby further enhancing electron‐ion synergy [63].

This reduction in bandgap may be attributed to increased grain size, optimized grain boundaries, and enhanced film density resulting from gradient doping, consistent with the previously observed FESEM results. Combined with FESEM analysis, it is evident that gradient films with uniform grains and smooth surfaces promote uniform electron‐hole mobility, thereby influencing the red shift phenomenon of the optical absorption edge. Furthermore, the gradient structure may optimize internal stress distribution within the film, enhancing optical stability. The FESEM observation of surface grains in gradient films and the reduced optical bandgap explain their superior performance compared to uniformly doped films: enhanced CE, accelerated response speed, and sustained high modulation depth during cycling.

**FIGURE 4:**
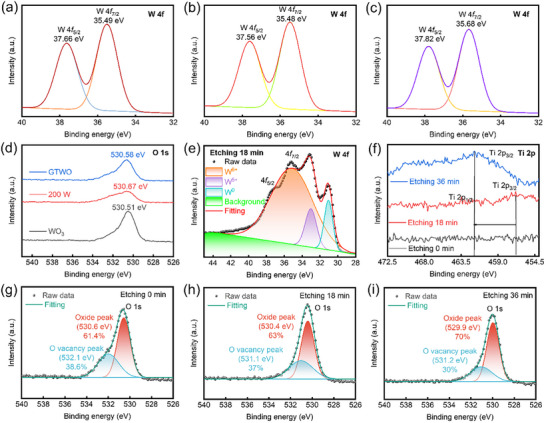
Surface W 4f spectra for different films of (a) pure WO_3_, (b) uniformly doped TWO (200 W RF power of TiO_2_ target) and (c) GTWO. (d) Surface O1s spectra comparison for pure WO_3_, uniformly doped Ti–WO_3_ (200 W RF power of TiO_2_ target), and GTWO. (e) Depth‐profile W 4f spectra after 18 min etching for the GTWO film, showing contributions from W^6+^ and W^5+^. (f) Ti 2p spectra at different etching times (0, 18, 36 min) indicating Ti diffusion for the GTWO film. O1s spectra and peak fitting for the GTWO film at etching times of (g) 0 min, (h) 18 min, and (i) 36 min.

XPS examination was performed on undoped, TWO (200 W RF power of TiO_2_ target), and GTWO films to determine the binding energy and oxidation state of the prepared samples. Figure S5 displays the XPS spectra of the three film surfaces. Figure 4a–c display the W 4f core‐level XPS spectra of the three film surfaces. XPS analysis indicates that W exists in its highest oxidation state (W^6+^), with no other oxidation states detected. This conclusion is consistent with previous literature reports [27]. The W 4f orbitals exhibit a double peak structure, with the W^6+^ 4f_5/2_ and W^6+^ 4f_7/2_ energy levels in the WO_3_ film located at binding energies of 37.66 and 35.49 eV, respectively, and a spin‐orbit splitting of 2.17 eV. In the uniformly doped WO_3_ film, the W^6+^ 4f_5/2_ and W^6+^ 4f_7/2_ levels are located at binding energies of 37.56 and 35.48 eV, respectively, with a spin‐orbit splitting of 2.08 eV. In the GTWO film, the W^6+^ 4f_5/2_ and W^6+^ 4f_7/2_ levels are located at binding energies of 37.82 and 35.68 eV, respectively, with a spin‐orbit splitting of 2.14 eV, consistent with the findings of Khan et al. [44]. Further observation reveals that the full width at half maximum (FWHM) of the W 4f peak in the doped WO_3_ film is broader and its intensity is lower compared to the pure WO_3_ film (Figure S6). This may be attributed to the weak Ti 3p peak embedded within the W 4f peak [64]. However, since the O 1s peak contributed by the W^6+^ bonded oxygen is located at approximately 530.8 eV, no peak position shift was observed among the three films within the permissible error range. The O 1s spectra of all three films exhibit distinct peaks corresponding to oxygen atoms (O_2_) participating in strong W═O bonding (Figure 4d). As shown in Figure 4e, we employed depth profiling to analyze the intermediate layer of the gradient‐doped film. By fitting the spectrum to three Gaussian peaks, the relative abundances of different valence states of tungsten (W^6+^, W^5+^, and W^0^) were compared based on integrated areas. It is evident that metallic tungsten is enriched in the gradient film compared to uniformly doped films. These W^0^ absorb more incident light, explaining why the gradient‐doped film exhibits lower transmittance [20, 32]. Simultaneously, the W 4f spectrum exhibits distinct valence state evolution, with the transformation from W^6+^ to W^5+^ and W^0^ revealing oxygen vacancy enrichment and enhanced reductive properties within the gradient‐doped film. This multivalency coexistence structure not only reflects oxygen distribution inhomogeneity but also provides favorable conditions for electron‐ion coupling and band continuity. High‐resolution scanning mode conducted an in‐depth analysis of peak shapes in GTWO films with varying etching durations (Figure 4f). The depth distribution of Ti elements obtained from XPS depth profiling is shown in Figure S7. Because the surface was not etched, the presence of surface carbon contamination, together with the surface‐sensitive nature of XPS, leads to a dilution effect on the atomic percentages, resulting in apparently lower W and Ti contents at the surface. After removal of the contamination layer, the intrinsic elemental distribution of the film becomes clearly observable. Ti elements appeared after 18 min of etching, likely due to increased sputtering power from the TiO_2_ target at this point, while the W metal target decreased, making the Ti presence more pronounced. After 36 min of etching, the Ti concentration increased, consistent with the designed top‐rich Ti gradient structure. Meanwhile, the Ti 2p_3/2_ peak shifted toward higher binding energy. At the bottom of the EC layer, Ti doping facilitates electron injection, optimizes band alignment, enhances interfacial stability, and provides additional ion migration pathways, thereby improving overall electrochemical performance and cycling stability [65]. Figure S8 shows the Ti 2p_3/2_ and W 4f spectra of TWO after 18 min of etching. Compared with GTWO, the W 4f peaks of TWO shift toward lower binding energy, indicating a more reduced chemical environment and a lower oxidation state of W. In contrast, the gradient structure of GTWO effectively maintains a higher oxidation state of W, while enhancing the synergistic effects of Ti–O bonding and oxygen‐vacancy distribution, keeping the W 4f peaks at relatively higher binding energy. This demonstrates that gradient doping can more effectively modulate the internal chemical environment and band structure of the film, enhancing electron transport, ion migration, and overall electrochemical performance, showing clear advantages over uniform doping. Figure 4g–i present depth‐profiled O 1s fitting results for gradient samples (etching 0/18/36 min). The O 1s spectrum is resolved into lattice oxygen (Oxide, ≈529.9–530.6 eV) and a high‐energy component (≈531.1–532.1 eV, labeled “O vacancy” in the figure).

Quantitative fitting indicates that the relative area of the high‐energy component decreases from ∼38.6% (0 min) to ∼30% (36 min) with etching time, while the lattice oxygen fraction increases from ∼61.4% to ∼70%. This trend indicates that the sample surface initially contained a higher proportion of high‐energy components (potentially hydroxyl/adsorbed oxygen or surface defects). As the surface layer was etched away, the spectrum revealed increased signals originating from lattice oxygen. Combined with the W 4f etching profile (enhanced low‐valent W after etching), we infer that the O 1s high‐energy component includes significant surface adsorption contributions, while the etched‐exposed interior regions exhibit genuine lattice oxygen and reduction‐induced low‐valent W (W^5+^/W^0^). Thus, Ti substitution doping regulates oxygen vacancy concentration distribution at different depths: the low oxygen vacancy region near the substrate significantly enhances film compactness and interfacial bonding strength, while moderate oxygen vacancies in the surface layer promote reversible Li^+^ insertion/extraction. This achieves high coloring efficiency and fast response while maintaining excellent cycling stability.

To further elucidate the physical origin of the enhanced electrochromic kinetics, the electronic energy levels of the GTWO films were characterized. The Eg determined from Tauc plots (Figure S9a) shows a systematic widening from 3.36 eV at the bottom layer to 3.40 eV at the top layer. Further investigation via UPS (Figure S9b, c) reveals that the work function (W.F.) increases from 5.25 eV (Bottom) to 5.35 eV (Top). Combined with the valence band (V.B.) edges (3.17–3.23 eV below Fermi Energy Level), the complete energy band alignment was established (Figure S9d). The spatial gradient in W.F. and Eg leads to a spontaneous band bending across the 330 nm thick film, generating an internal built‐in electric field. Unlike conventional uniform doping, which primarily improves isotropic conductivity, this directionally built‐in electric field provides an additional electromotive force that facilitates the transport of Li^+^ ions and electrons. This mechanism effectively “decouples” the kinetic enhancement from simple defect‐induced conductivity, explaining the simultaneous improvement in coloration efficiency and switching speed.

**FIGURE 5:**
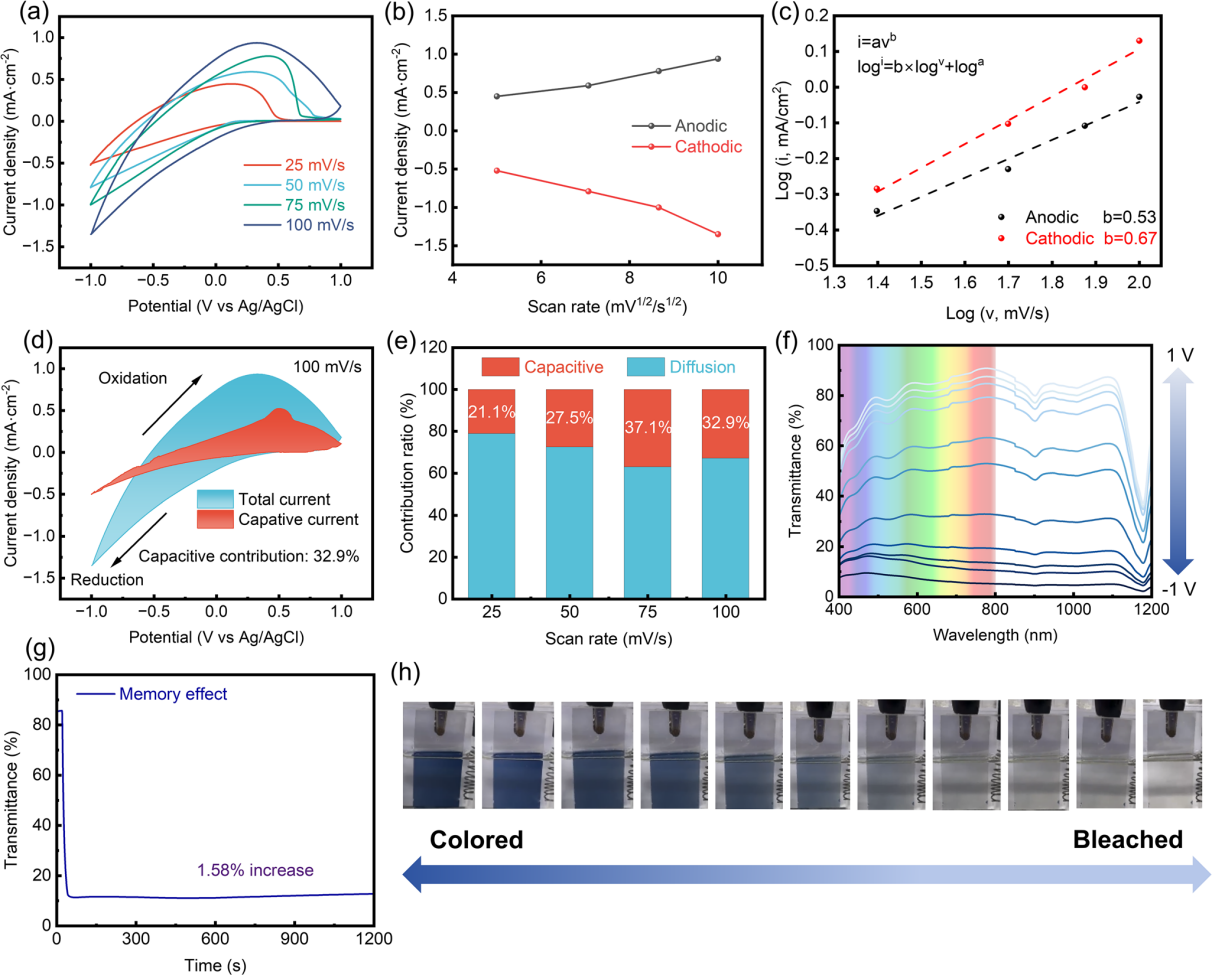
Electrochemical and optical property analysis of GTWO films: (a) CV curves at different scan rates. (b) Relationship between oxidation/reduction peak current density and the square root of scan rate. (c) log (*i*) −log (*v*) fitting analysis. (d) Schematic of capacitive contribution separation. (e) Bar chart showing the ratio of capacitive/diffusive contributions at different scan rates. (f) Transmission spectra at different voltages. (g) Images showing the film's coloration from −1 V to bleaching at +1 V. (h) Memory effect test.

The potential mechanism underlying the exceptional electrochemical performance of the GTWO film was further investigated. CV curves in Figure 5a reveal distinct redox peaks at various scan rates, corresponding to the insertion and deinsertion of Li^+^ from WO_3_. Moreover, the enclosed area of the CV curves increases with rising scan rates, indicating pseudocapacitive behavior in the films [9]. As the scanning rate increases, the redox peaks gradually broaden, and the peak potentials shift toward both positive and negative directions, indicating that Li^+^ diffusion becomes progressively restricted and surface capacitive processes become more pronounced [52]. The charge obtained from CV curves may originate from surface‐dominated non‐Faradaic reactions (EDLC behavior), Faradaic reactions with surface contributions (pseudocapacitive behavior), and diffusion‐dominated rapid Faradaic reactions [56, 62]. Fitting the linear slope of the peak current (*i_p_*) against the square root of the scan rate ($v^{\frac{1}{2}}$) reveals the ion diffusion behavior of the electrode (Figure 5b). A linear relationship between *i_p_* and $v^{\frac{1}{2}}$ was observed, indicating the material's ion diffusion characteristics. The ion diffusion coefficient (D) was determined using the Randles–Sevcik Equation [9, 11, 66]:

(5) $$D^{1/2}=i_{p}\;/\left(2.72\times 10^{5}\times n^{\frac{3}{2}}\times A\times C\times v^{\frac{1}{2}}\right)$$

Here, *i_p_*, n、A, C, and *v* represent the peak current density, number of electrons, working electrode area, electrolyte ion concentration, and scan rate of the CV curve, respectively. The value of *i_p_* can be obtained from Figure 5a. In this work, n is taken as 1, corresponding to the single‐electron transfer involved in the Li^+^ insertion/extraction process of WO_3_‐based electrochromic reactions. The effective working electrode area A is defined by the geometric area of the electrochromic film exposed to the electrolyte (≈ 5.4 cm^2^). The electrolyte ion concentration C is fixed by the composition of the Li^+^‐based electrolyte used in the measurements (≈ 0.5 m). The scan rate *v* is directly obtained from the CV testing conditions and is specified in the corresponding figure captions (25, 50, 75, and 100 mV s^−1^). For the gradient‐doped film, the D values during the coloring/decoloring process are 1.52 × 10^−8^ and 4.0 × 10^−8^ cm^2^ s^−1^, respectively. These values are significantly higher than those of pure WO_3_ film, which typically range between 10^−9^ and 10^−10^ cm^2^ s^−1^ [9, 67]. This enhancement effect stems from the optimization of the film's microstructure and ion transport pathways achieved by the gradient‐doped structure [68]. Additionally, the area enclosed by the CV curve reflects the combined contribution of diffusion‐controlled processes and surface capacitance effects to the overall charge storage capacity. We conducted a 2D analysis of the electrochromic mechanism. First, by fitting CV curves using the following empirical equation, we can separately estimate the contributions of capacitive and diffusion effects to electrochromic behavior. This equation enables a precise understanding of the charge storage process, defined as [62, 69]:

(6) $$i=\;av^{b}$$

where *i* is the peak current, *v* is the scan rate, and a and *b* are variable parameters. By analyzing the slope of the log (*i*) versus log (*v*) curve, the *b* value describing the type of charge storage process can be determined. Ideally, *b* = 1 for capacitive‐controlled processes, *b* = 0.5 for diffusion‐controlled processes, and *b* values for pseudocapacitive processes should lie between 0.5 and 1. Using Equation (6), we calculated b values of 0.67 and 0.53 for cathodic and anodic contributions, respectively, at different scan rates for the gradient‐doped film. This indicates that the electrochemical behavior of the film is both diffusion‐controlled and exhibits significant pseudocapacitive contributions (Figure 5c). The Dunn method was employed to quantify the relative contributions of diffusion‐controlled processes and capacitive effects. The explicit contribution ratios of different charge‐discharge mechanisms were considered via Equation (7) [62, 69]:

(7) $$i\;\left(V\right)=k_{1}\;v+k_{2}v^{0.5}$$

Here, *i*(*V*) represents the instantaneous current. *k*_1_ and k_2_ are derived from the slope and vertical intercept (as *v*→∞), respectively. *k*_1_ *v* and k_2_ *v* ^0.5^ denote capacitive and ionic diffusion behaviors, respectively. At a scan rate of 100 mV s^−1^, the capacitive contribution (red region) accounts for 37.1% of the total charge storage (Figure 5d). Furthermore, Figure 5e demonstrates that the charge‐discharge process of the gradient film is predominantly governed by ionic diffusion behavior, accounting for over 62% of the contribution. When the scan rate increased from 25 to 100 mV s^−1^, the capacitive contribution rose from 21.1% to 37.1% (Figure 5e and Figure S10). This indicates that the gradient‐doped structure optimizes the pathways for ion insertion/extraction, effectively enhancing the film's responsiveness at high scan rates while maintaining excellent electrochemical reversibility. When the scan rate increased from 25 to 100 mV s^−1^, the diffusion contribution decreased by only 20.28%. Moreover, the diffusion‐controlled process in the gradient‐doped film remained the dominant contributor at high scan rates, demonstrating the excellent rate performance of the gradient film [52]. In terms of optical performance, the transmission spectrum (Figure 5f) demonstrates that the gradient‐doped film exhibits outstanding optical modulation capabilities across the visible to near‐infrared spectrum. The film displays dynamically continuous transmittance and color‐tuning characteristics under varying operating voltages (Figure 5g). In its initial state, the gradient film exhibits high transmittance, with an average transmission exceeding 78% in the 400–800 nm wavelength range. When the voltage rises to −0.2 V, the color shifts to light blue, and at −1 V, it changes to dark blue, with excellent color uniformity. The film's color‐changing process is demonstrated in the supplementary video. A 20 min memory effect test (Figure 5h) shows minimal transmittance variation (<2%), further validating the stability of its optical performance. Combined with the preceding SEM analysis, the uniform grain distribution and dense structure of the gradient film not only contribute to a slight red shift in the optical absorption edge but also significantly enhance the electrochemical response rate and cycling stability.

**FIGURE 6:**
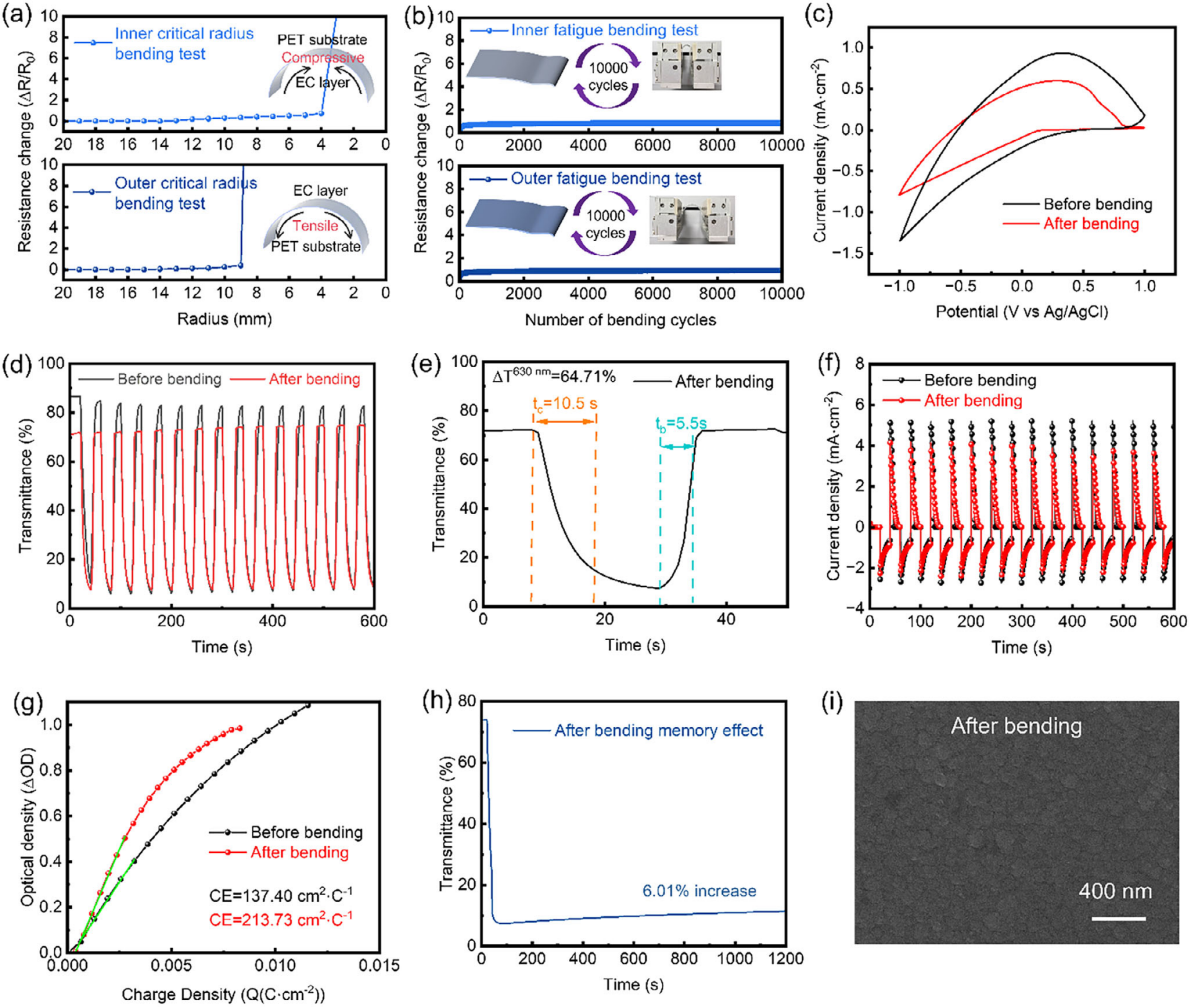
Comparison of electrical and optical properties of GTWO films before and after bending: (a) Critical radius bending tests on inner and outer sides, (b) Fatigue bending cycle tests (10 000 cycles) on inner and outer sides, (c) CV curves before and after bending, (d) Transmittance versus time curves at 630 nm before and after bending, (e) Transmittance modulation cycles before and after bending, (f) Chronoamperometric curves before and after bending, (g) CE fitting curves calculated from the optical density change versus charge density, (h) Memory effect test after bending, (i) FESEM image of surface morphology after bending.

Figure 6 demonstrates the structural stability and changes in electrochemical/optical properties of GTWO films under bending stress. As shown in Figure 6a, which presents critical bending radius measurements for inner and outer bending, the film exhibits virtually no significant resistance change until the inner bending radius is reduced to approximately 4 mm (outer bending radius: 9 mm), indicating excellent flexibility. The critical bending radius is a key metric for evaluating mechanical stability, as it defines the minimum curvature a flexible electrochromic film can withstand without irreversible electrical degradation, thereby directly reflecting its mechanical robustness under practical deformation conditions. Notably, even when repeatedly bent to its critical radius for 10 000 cycles, the device exhibits an extremely low resistance variation of less than 1% (Figure 6b), which underscores its exceptional fatigue resistance and mechanical robustness. To further clarify the influence of the gradient Ti‐doping structure on the mechanical stability of the films, systematic bending tests were conducted on the uniformly TWO films under identical conditions, and the results were comparatively analyzed with those of the GTWO films. As shown in Figure S11a, the TWO film exhibits a distinct failure threshold during the critical bending radius test. Under inner bending conditions, a sharp increase in resistance is observed when the bending radius is reduced to approximately 3 mm, corresponding to a critical bending radius of about 4 mm. Under outer bending conditions, the critical bending radius is around 10 mm. These results indicate that irreversible microcracks or interfacial damage begin to develop within the TWO film even at relatively large bending radii.

Further fatigue bending tests reveal that the resistance variation of the TWO film gradually accumulates during 10 000 repeated bending cycles. In particular, under inner bending conditions, ΔR/R_0_ continuously increases, indicating limited structural stability under cyclic mechanical strain. Under outer bending conditions, ΔR/R_0_ exceeds 2 after only a relatively small number of bending cycles, reflecting pronounced resistance degradation at an early stage. In contrast, the GTWO film exhibits significantly smaller resistance drift and a more stable fatigue response under the same testing conditions, demonstrating the clear advantage of the gradient structure in suppressing the accumulation of mechanical damage.

The enhanced mechanical stability can be attributed to the continuous compositional transition induced by gradient Ti doping along the thickness direction, which effectively mitigates localized stress concentration arising from bending deformation and reduces the initiation and propagation of cracks either within the film or at the film/substrate interface. Consequently, compared with the uniformly TWO film, the GTWO film exhibits superior flexibility while maintaining stable electrochromic functionality, further confirming the engineering advantages of gradient‐doped architectures for flexible electrochromic devices.

While the ∼10% increase in CE compared to the TWO uniform films may appear incremental, its significance must be evaluated alongside the mechanical durability. As shown in the bending tests (Figure S11b), the TWO film exhibited significant performance degradation due to micro‐crack propagation. In contrast, the gradient‐Ti distribution effectively redistributes the interfacial strain, allowing the film to sustain high coloration efficiency even under severe mechanical deformation. This suggests that the gradient structure successfully breaks the efficiency‐flexibility trade‐off commonly observed in doped WO_3_ systems.

To validate the device's mechanical endurance, we also tested the electrochemical performance and dynamic optical transmittance spectrum at 630 nm wavelength of the gradient electrochromic film layer after 500 bending cycles (Figure 6c, d). Results show that the CV curve area and chronoamperometric response slightly decreased post‐bending, yet retained stable redox characteristics. As shown in Table 2, the film maintained 82% of its pre‐bending transmittance modulation after bending, still exhibiting a high transmittance modulation amplitude (ΔT_630_ nm ≈ 64.71%). This indicates that it retains excellent stability even under compressed conditions. Granqvist [70] noted in his work that CE depends on “the charge effectively participating in the optical response,” rather than the total charge amount. When mechanical stress causes partial failure of active regions or channels, the total charge insertion may decrease (resulting in reduced CV area), but the remaining regions exhibit higher charge utilization. This manifests as enhanced optical density change per unit charge, i.e., an increase in CE [71]. Furthermore, research on flexible ECD indicates that bending stress often reduces optical modulation amplitude, yet it can still maintain or even enhance charge utilization efficiency to a certain extent [72]. For example, Li et al. [66]. reported that the ΔT of TiO_2_/MXene flexible devices slightly decreased after bending, while charge utilization efficiency remained satisfactory. The reduced transmittance of the bent film in the bleached state may indicate local crack closure, pore shrinkage, or densification effects during bending or cycling, leading to increased scattering or absorption of light during transmission. Concurrently, localized stress may also cause increased interfacial roughness, thereby reducing overall transmittance [64]. After 500 bending cycles at a 4‐mm bending radius, the t_c_/t_b_ of the flexible device changed from 11/11.5 s to 10.5/5.5 s (Figure 6e, f, Table 2), indicating a slight reduction in switching time and a significant acceleration of the bright ion migration process. Collectively, these results demonstrate that the GTWO‐based electrochromic layer exhibits outstanding mechanical flexibility and cycling stability, offering broad application prospects in flexible displays, lightweight smart windows, and wearable integrated systems. Notably, in the CE comparison (Figure 6g), the CE increased from 137.4 to 213.73 cm^2^ C^−1^ after bending (same GTWO). From a physical perspective, electron–ion coupling is generally expected to be optimized in structurally continuous and defect‐minimized films. Mechanical bending may introduce microcracks or structural discontinuities, which could potentially disrupt electronic percolation pathways. Therefore, the improved CE observed after deformation is more plausibly attributed to increased electrolyte infiltration and enhanced interfacial accessibility caused by bending‐induced microcracks. Such structural features may shorten ion diffusion distances and promote surface‐dominated electrochemical reactions, leading to an apparent increase in CE [73]. It has been widely reported that nanostructured or porous WO_3_ films exhibit higher coloration efficiency due to shortened Li^+^ diffusion paths and increased active surface area [24]. Figure 6h of the memory effect test shows that the transmittance change improved by only approximately 6.01%, further verifying the optical stability of the device after bending. FESEM image (Figure 6i) reveals that the surface morphology of the bent film became denser and more uniform after compression, consistent with the reduced transmittance results. No significant cracks or delamination were observed, demonstrating its excellent structural toughness.

**TABLE 2:** Electrochromic parameters of GTWO/IGTO/PET films before and after 500 bending cycles, including bleached transmittance, colored transmittance, ΔT, t_c_, t_b_, and CE.

Samples	Bleached Transmittance [%]	Colored Transmittance [%]	Color Modulation [%]	Bleached Time [s]	Colored Time [s]	Coloration Efficiency [cm^2^ C^−1^]
Before bending	84.91	6.00	78.91	11	11.5	137.40
After bending	72.17	7.46	64.71	10.5	5.5	213.73

As shown in Figure S12, the electrochemical behavior, electrochromic response, coloration efficiency, and surface morphology of the TWO thin film before and after bending were systematically investigated to evaluate its structural stability and functional evolution under mechanical deformation. As illustrated in Figure S12a, after bending, the CV curves of the TWO film exhibit a pronounced decrease in redox peak current density and integrated area, indicating a significant reduction in the overall charge injection/storage capability induced by mechanical bending. Meanwhile, noticeable distortion of the CV curve shape is observed, suggesting that the uniform TWO film is more susceptible to structural damage during bending, leading to deteriorated electrochemical reversibility. Figure S12b presents the dynamic transmittance variation of the TWO film before and after bending. It can be clearly seen that the optical modulation amplitude decreases markedly after bending, accompanied by increased transmittance fluctuations during repeated coloration/bleaching cycles. This behavior implies that mechanical deformation adversely affects ion transport and the stability of the optical response in uniform TWO films. Furthermore, as shown in Figure S12c, the ΔOD–Q relationship of the pristine TWO film exhibits good linearity, corresponding to a relatively high coloration efficiency (CE = 125.03 cm^2^ C^−1^). However, after bending, the slope of this linear relationship decreases significantly, and the CE drops to 67.91 cm^2^ C^−1^. This result indicates that mechanical deformation not only reduces the amount of injectable charge but also lowers the utilization efficiency of injected charges for optical modulation. SEM images in Figure S12d reveal the presence of pronounced through‐thickness cracks on the surface of the bent TWO film. These cracks disrupt the continuous and compact film structure, leading to broken electron transport pathways and non‐uniform ion migration channels, which collectively result in a substantial deterioration of charge storage capability and electrochromic response efficiency.

Notably, compared with the GTWO films prepared in this work, the uniform TWO film exhibits much more severe performance degradation after bending. This comparison suggests that uniform TWO films are more susceptible to structural disruption under mechanical perturbation, which may adversely affect electrochemical stability. In contrast, the compositional gradient in GTWO films appears to mitigate deformation‐induced damage, thereby preserving a more stable electrochemical response and coloration performance.

**FIGURE 7:**
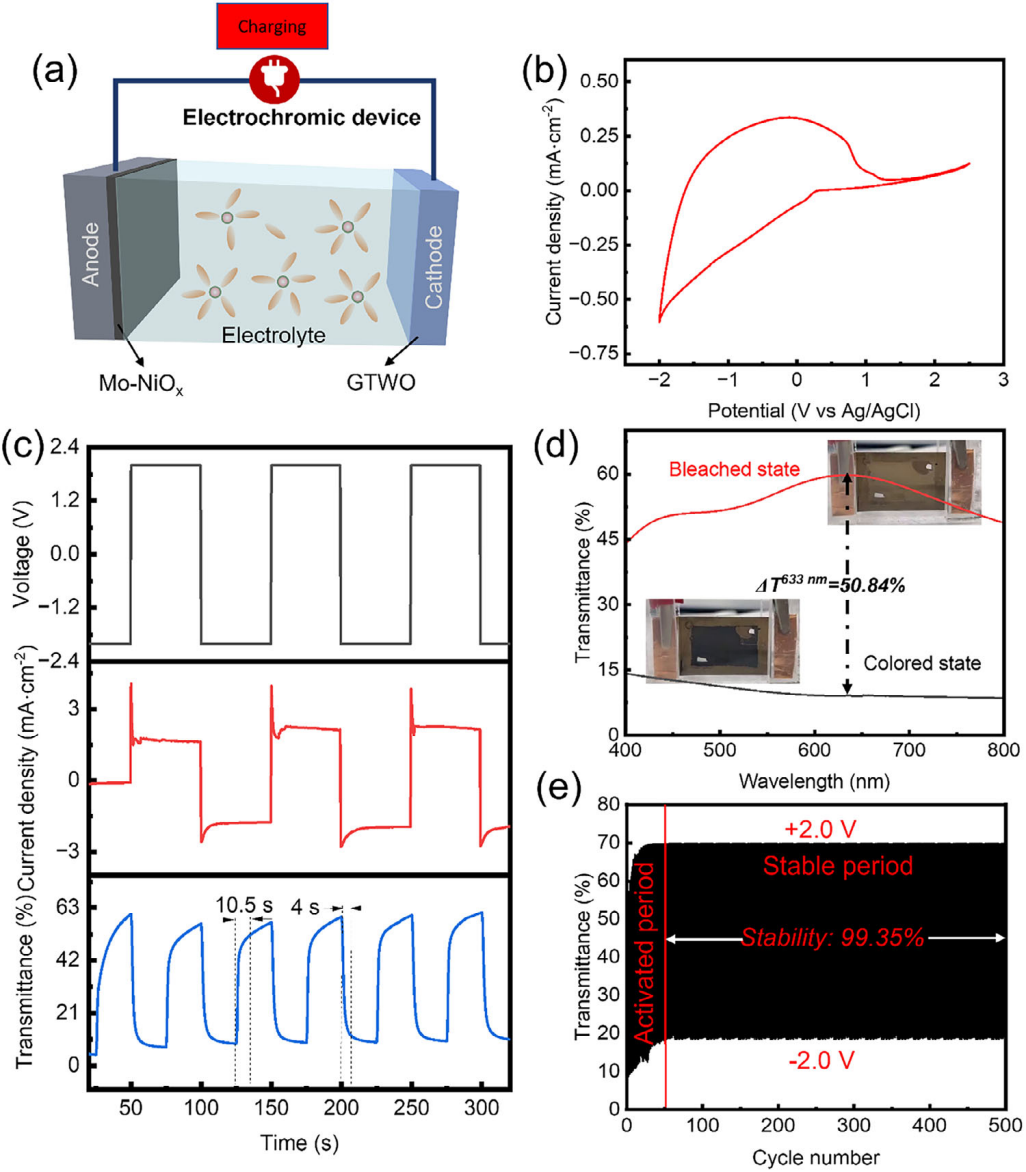
The structure and performance characterization of GTWO‐based ECD: (a) Schematic diagram of the device structure, (b) CV curves, (c) While the optical modulation for applied voltage of −2 V for colored state and 2 V for bleached state within interval of each step was controlled at 50 s; corresponding CA curve of ECD; switching time between bleached and colored states measured at 633 nm between 2 and −2 V, (d) Transmittance spectra and optical pictures of the optimized ECD in the colored and bleached states for the 400–800 nm range, (e) The durability of ECD in terms of transmittance optical modulation at 633 nm following 500‐cycles.

After characterizing the electrochemical performance and optical properties of the GTWO EC films, it is necessary to further evaluate the performance of the optimized GTWO EC films in actual devices. By assembling the prepared high‐performance EC films into Mo–NiO_x_/GTWO devices, the optical modulation capability, electrochemical reversibility, and cycling stability of the films under realistic working conditions can be directly observed, thereby validating the effectiveness of the film design and performance optimization. Figure 7 presents the structure and performance characterization of the fabricated devices. Figure 7a shows a schematic diagram of the device, in which the GTWO film serves as the cathodic EC layer, achieving controllable coloration and bleaching through reversible Li^+^ insertion/extraction, while the Mo–NiO_x_ anode provides charge compensation to ensure balanced electron and ion transport during cycling. CV measurements (Figure 7b) reveal distinct and symmetric oxidation/reduction peaks within a ± 2 V voltage range, indicating that the GTWO film exhibits highly reversible electrochemical behavior. Dynamic switching performance tests (Figure 7c) show a coloration time of approximately 4 s and a bleaching time of about 10.5 s, with a transmittance modulation ΔT_633 nm_ reaching 50.84% (Figure 7d), demonstrating excellent optical modulation capability. Figure S13 presents optical photographs of the electrochromic device recorded at different applied voltages ranging from −2.0 to +2.0 V. As the bias voltage is swept from negative to positive values, the device undergoes a continuous and reversible color transition from a dark‐colored state to a bleached state. The gradual evolution of optical appearance indicates a stable and controllable electrochromic response under different operating voltages. No noticeable color residue or optical degradation is observed during the coloration–bleaching process, suggesting good electrochemical reversibility and uniform ion/electron transport within the device. As illustrated in Figure 7, an operating voltage of ±2.0 V was applied to drive the ECD. This voltage was selected to achieve an optimal balance between optical modulation depth and switching kinetics. While the GTWO material can initiate coloration at lower potentials, the ±2.0 V driving force ensures rapid ion migration across the gradient interface, fully utilizing the material's capacity without exceeding the electrochemical stability window of the electrolyte. This is evidenced by the stable cycling performance shown in Figure 7e, confirming that no irreversible side reactions occur at this potential. Long‐term cycling tests (Figure 7e) indicate that the device maintains stable transmittance changes even after 500 cycles, confirming good cycling stability and practical application potential. Overall, the fabricated Mo–NiO_x_/GTWO device not only reflects the rapid response and high optical modulation performance achieved through optimized film design but also provides a reliable experimental basis for the development of practical ECD.

By comparing with existing literature, the ECD developed in this work highlights a superior balance of performance (Table 3): it achieves a response time of 14.5 s and color modulation of 50.84%, while maintaining a highly competitive coloration efficiency of 194.48 cm^2^ C^−1^ (Figure S14) and exceptional cycling stability with 99.35% retention after 500 cycles. This demonstrates that regulating crystal growth and ion migration pathways through gradient structures can significantly improve the performance of electrochromic films. It provides a viable strategy for designing high‐performance, long‐lifetime electrochromic devices and offers a reference for applying gradient structures in other functional films.

**TABLE 3:** Comparison of electrochromic properties for different ECD configurations.

ECD Configuration	EC Properties			
	Color Modulation	Response Time [s]	Coloration Efficiency [cm^2^ C^−1^]	Cycle Stability
ITO/W‐NiO/LiTaO_3_/WO_3_/ITO [74]	41.7%	61.6	—	Slowly goes down after 70 cycles
ITO/WO_3_/LiNbO_3_/Al‐LiNiOx/ITO [75]	44%	22	55	10000 (77.8%)
ITO/WOx/Electrolyte/PW/ITO [1]	57%	36	104.98	No decrease after 23000 s
ITO/Q‐PHI WO_3_ Electrolyte/NiO/IGTO [69]	67.6%	29.6	354.4	1000 (65.8%)
Porous a‐WO_3_ – based ECD [24]	86.2%	21.7	67.1	—
WO_3_/W/Au/Nylon 66 /Au [76]	—	4.76	—	100 (97.83%)
WO_3_/ TiO_2_ – based ECD [38]	69%	41	69	1000 (No significant change)
ITO/V2DP/ Electrolyte/ITO [77]	25%	4	989	200 (95.6%)
IGTO/GTWO/Electrolyte/ Mo–NiOx /IGTO (This work)	50.84%	14.5	194.48	500 (99.35%)

## 3 Conclusion

In this study, the effects of Ti uniform and gradient doping on the electrochromic and optical properties of WO_3_ films were systematically investigated. The results reveal that the GTWO film achieves a higher CE while maintaining a large ΔT, indicating that the gradient‐doped structure effectively enhances the electron–ion coupling efficiency. Structural and optical analyses demonstrate that the gradient‐doped structure promotes the formation of a uniform surface with low roughness, thereby facilitating more efficient ion insertion/extraction pathways. Moreover, Ti incorporation improves electron transport, resulting in a faster EC response and higher CE. Electrochemical characterization, including CV and chronoamperometry, further confirms that the GTWO film exhibits superior ion kinetics compared to the uniformly doped one. In addition, cyclic and mechanical endurance tests show excellent stability, with only a slight reduction in transmittance modulation under bending stress while retaining a high CE, suggesting that mechanical deformation mainly affects local active regions rather than overall charge utilization. Furthermore, the optimized GTWO films were assembled into ECD to evaluate their practical performance. The devices exhibited rapid coloration (∼4 s) and bleaching (∼10.5 s), a high transmittance modulation (ΔT_633 nm_ ≈ 50.84%), and excellent cycling stability over 500 cycles, demonstrating that the film design translates effectively into high‐performance device operation. Overall, the GTWO film combines the structural continuity of a homojunction with the energy band tunability of a heterojunction. This strategy provides an effective pathway for enhancing EC performance and offers new insights into the development of flexible and wearable electro‐optical devices.

## 4 Experimental Section

### 4.1 Materials and Preparation of the EC Layer/IGTO/PET

The preparation of the amorphous IGTO electrodes used in this experiment follows the methodology described in previous research [34]. On the IGTO electrodes, a TWO EC layer was obtained by simultaneously sputtering a W metal target via direct current (DC) and a TiO_2_ target via reactive frequency (RF) magnetron sputtering. First, Ti doping was achieved by varying the RF power of the TiO_2_ target (0, 100, 150, and 200 W) while maintaining the W metal target at 200 W DC power under conditions of Ar:O_2_ = 49/21 and a working pressure of 10 mTorr. To ensure a fair comparison, the sputtering time was adjusted at different TiO_2_ RF powers to maintain a comparable film thickness of ∼330 nm for all TWO films (Table S1). TiO_2_ and W metal targets (Dasom, 99.99% purity) were pre‐sputtered for 5 min to remove contaminants on IGTO surfaces. Ar gas (99.999% purity) and O_2_ gas (99.999% purity) were used as sputtering gases. Subsequently, a film with a gradient distribution of Ti was designed, where the Ti content decreases from bottom to top. No etching process was used in this work. The film patterning was achieved by a shadow‐masking approach using adhesive tape to cover the undesired regions prior to sputtering deposition. After deposition, the tape was removed to define the active area.

### 4.2 Characterization of WO_3_, TWO and GTWO

The sputtering rate was verified by performing thickness measurements using an Alpha‐step 250 profilometer. Further characterization of the surface morphology and structural features of the EC layer was conducted using field emission scanning electron microscopy (FESEM, JSM‐7600F), X‐ray diffraction (XRD, Smart Lab, Shimadzu Corporation), and X‐ray photoelectron spectroscopy (XPS, PHI 5000 VersaProbe). XPS depth profiling was performed using a 3 kV ion gun with an etching rate of approximately 6.1 nm min^−1^, and high‐resolution spectra were collected at each etching depth to obtain the elemental distribution as a function of depth. To evaluate the mechanical and electrical stability of Ti‐gradient‐doped WO_3_ electrochromic films under stress conditions, stress was applied using a bending machine provided by Junil‐Tech, with real‐time strain monitoring. Concurrently, a custom‐designed dual‐mode internal/external bending test platform simulated the dynamic deformation process of flexible electrodes in actual wearable devices, systematically investigating the film's durability and performance retention. The electrochemical and optical properties of the TWO/IGTO/PET structure were evaluated using a UV–vis–NIR spectrophotometer (Jasco V‐770) and an electrochemical workstation (ZIVE SP2, WonATech) in a three‐electrode configuration. To ensure consistency with established literature in the electrochromic field, a bare IGTO‐coated PET substrate was employed as the optical reference. It should be noted that the initial transmittance of the as‐sputtered films (presented in Figure S3) differs from that of the bleached state (presented in Figure 1b). TWO/IGTO/PET was employed as the working electrode, with platinum (Pt) wire serving as the counter electrode and silver/silver chloride (Ag/AgCl) electrode as the reference electrode, selected for their stability in various aqueous electrolytes [78]. The electrolyte used was 0.5 M LiClO_4_ dissolved in a water solution of propylene carbonate (PC). Ultraviolet photoelectron spectroscopy (UPS) measurements were performed on GTWO films using a He I source (21.2 eV) to determine the work function and probe the built‐in electric field. To maintain high data integrity, electrochromic characterization followed the rigorous benchmarking protocols established for energy storage and conversion materials [79]. All reported values represent the average of five discrete device batches. The exceptional consistency in switching kinetics and coloration efficiency across these batches underscores the robustness and scalability of the gradient Ti‐doping strategy.

### 4.3 Fabrication and Analysis of ECD

The preparation of Mo–NiO_x_ was carried out following the preparation reported by Park et al. [80]. First, the previously synthesized GTWO films were used as the electrochromic cathode, whose gradient Ti‐doped structure provides high electron–ion coupling efficiency and excellent optical modulation performance. The Mo–NiO_x_ film served as the anodic charge‐compensating layer, ensuring balanced electron and ion transport during Li^+^ insertion and extraction. During device assembly, the GTWO cathode, Mo–NiO_x_ anode, and a liquid electrolyte composed of 0.5 M LiClO_4_ dissolved in a water solution of PC were stacked and sealed to form a complete ECD. This electrolyte not only provides reversible Li^+^ transport pathways but also ensures stable electrochemical conditions during cycling. The ECDs were assembled in a sandwich configuration (PET/IGTO/GTWO/Electrolyte/ Mo–NiO_x_ /IGTO/PET) with an active area of approximately 5.4 cm^2^. After assembly, the devices were characterized using an electrochemical workstation (ZIVE SP2, WonATech) and a UV‐Vis‐NIR spectrophotometer (Jasco V‐770) to evaluate optical performance (transmittance modulation ΔT and optical response speed), electrochemical behavior (CV and CA), as well as long‐term cycling stability. This procedure not only verifies the rapid response and high optical modulation capability of the GTWO films in devices but also provides a reliable experimental basis for optimizing the overall performance of ECDs.

### 4.4 Statistical Analysis

All electrochemical and electrochromic measurements were performed using at least three independently prepared samples to ensure reproducibility. Cyclic voltammetry, chronoamperometry, in situ optical transmittance measurements, and bending tests were repeated no fewer than three times for each sample. The reported results represent typical or averaged behaviors obtained from repeated measurements.

CE was determined from the linear fitting of the ΔOD–Q plots using identical fitting procedures for all samples. Linear fitting was carried out using OriginPro software. No data points were selectively excluded unless explicitly stated.

Morphological and structural characterizations, including SEM, XRD, and XPS analyses, were conducted on representative samples, and the observed trends were confirmed to be reproducible across independently prepared films.

## Conflicts of Interest

The authors declare no conflict of interest.

## Supporting information




**Supporting File 1**: smll73166‐sup‐0001‐SuppMat.docx.

**Supporting File 2**: smll73166‐sup‐0002‐VideoS1.mp4.

## Data Availability

The data that support the findings of this study are available from the corresponding author upon reasonable request.
