# Supplementary material for: Compositional Gradient–Engineered Ti–WO3 Films for Simultaneous Enhancement of Coloration Efficiency and Mechanical Robustness
Source: Small. 2026 Mar 23;22(27):e14826. doi: 10.1002/smll.202514826 (PMC13173321; doi:10.1002/smll.202514826)
Supplement: Supplementary file 1 — Supporting File 1: smll73166‐sup‐0001‐SuppMat.docx. [file SMLL-22-e14826-s002.docx]

**Compositional Gradient–Engineered Ti–WO₃ Films for Simultaneous Enhancement of Coloration Efficiency and Mechanical Robustness**

*Fang Luo^1^,* *Chang-Shin Park^1^, Yeoung-Eun Seo^1^, Xiaosong Jiang^2^ and Han-Ki Kim^1,3,^* *^*^*

^1^School of Advanced Materials Science and Engineering, Sungkyunkwan University, Suwon, Gyunggi-do 16419, Republic of Korea

^2^School of Materials Science and Engineering, Southwest Jiaotong University, Sichuan, Chengdu, 610031, China

^3^Department of Display Engineering, Sungkyunkwan University, Suwon, Gyunggi-do 16419, Republic of Korea

**Supporting information**


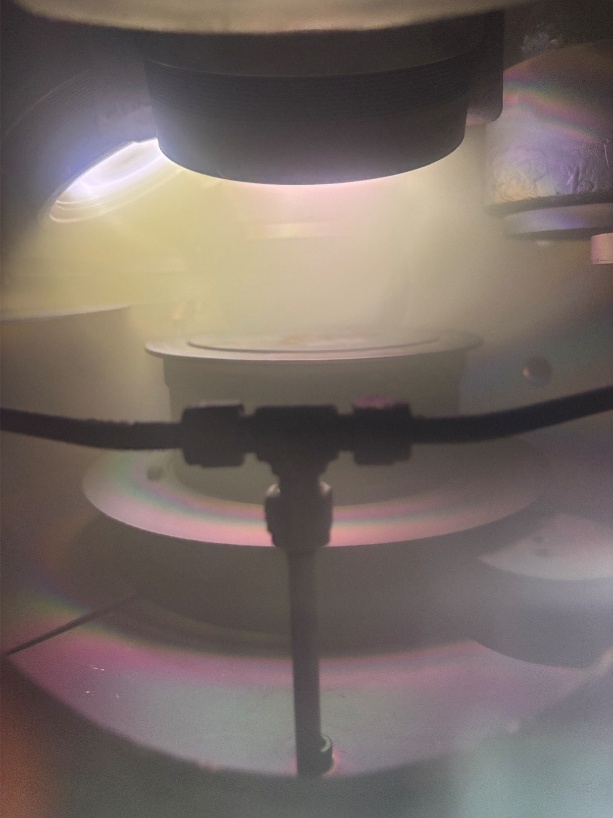


Figure 1. Actual picture of co-sputtering process.


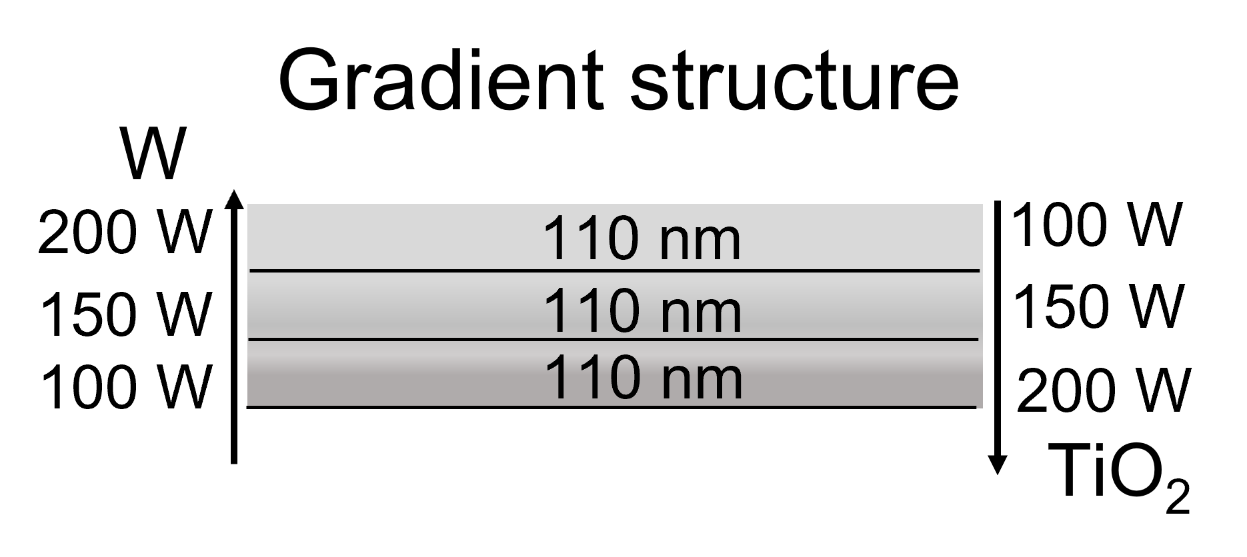


Figure 2. Schematic of GTWO film.


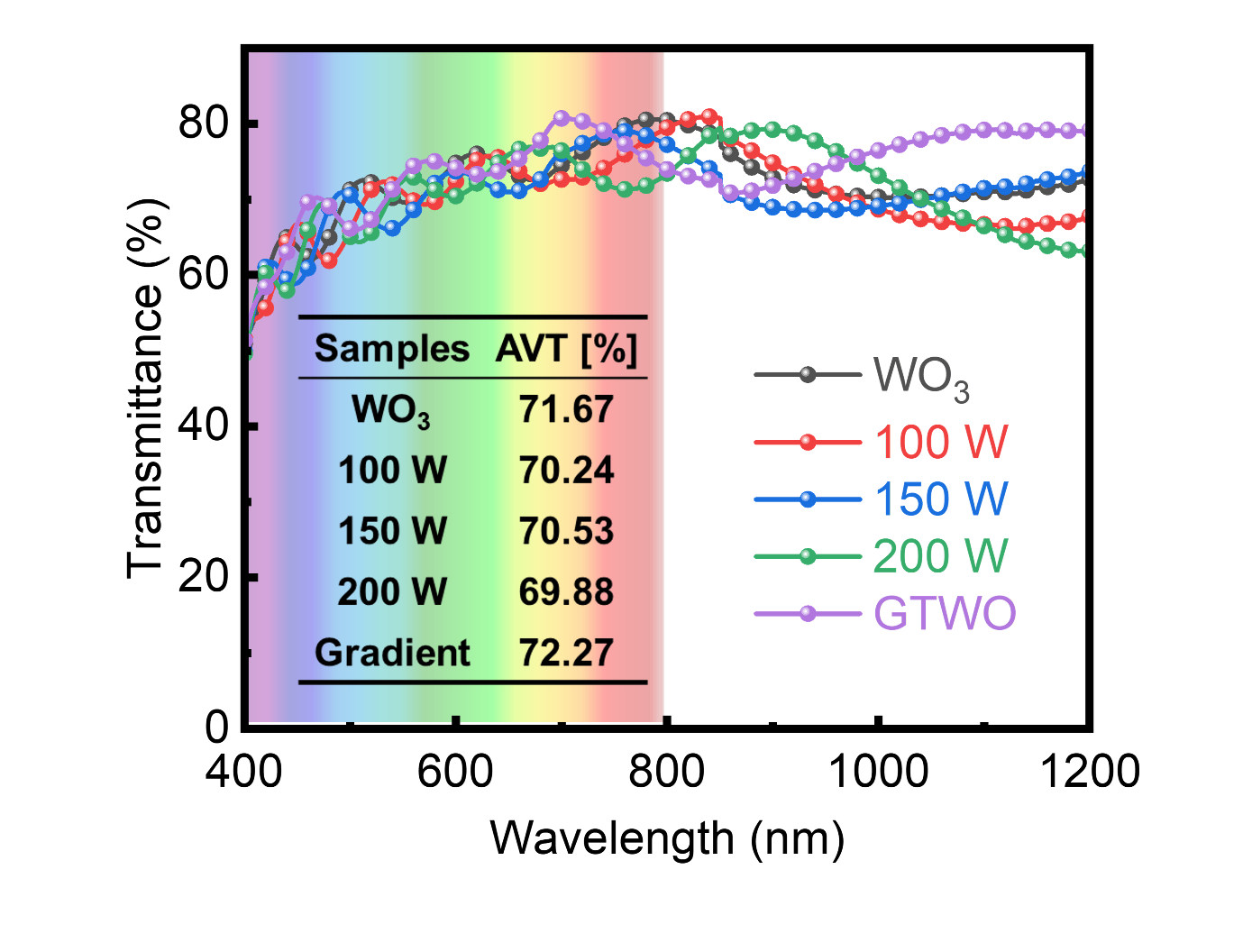


Figure 3. Optical transmittance spectra and average visible transmittance (AVT%) of different as-sputtered films.


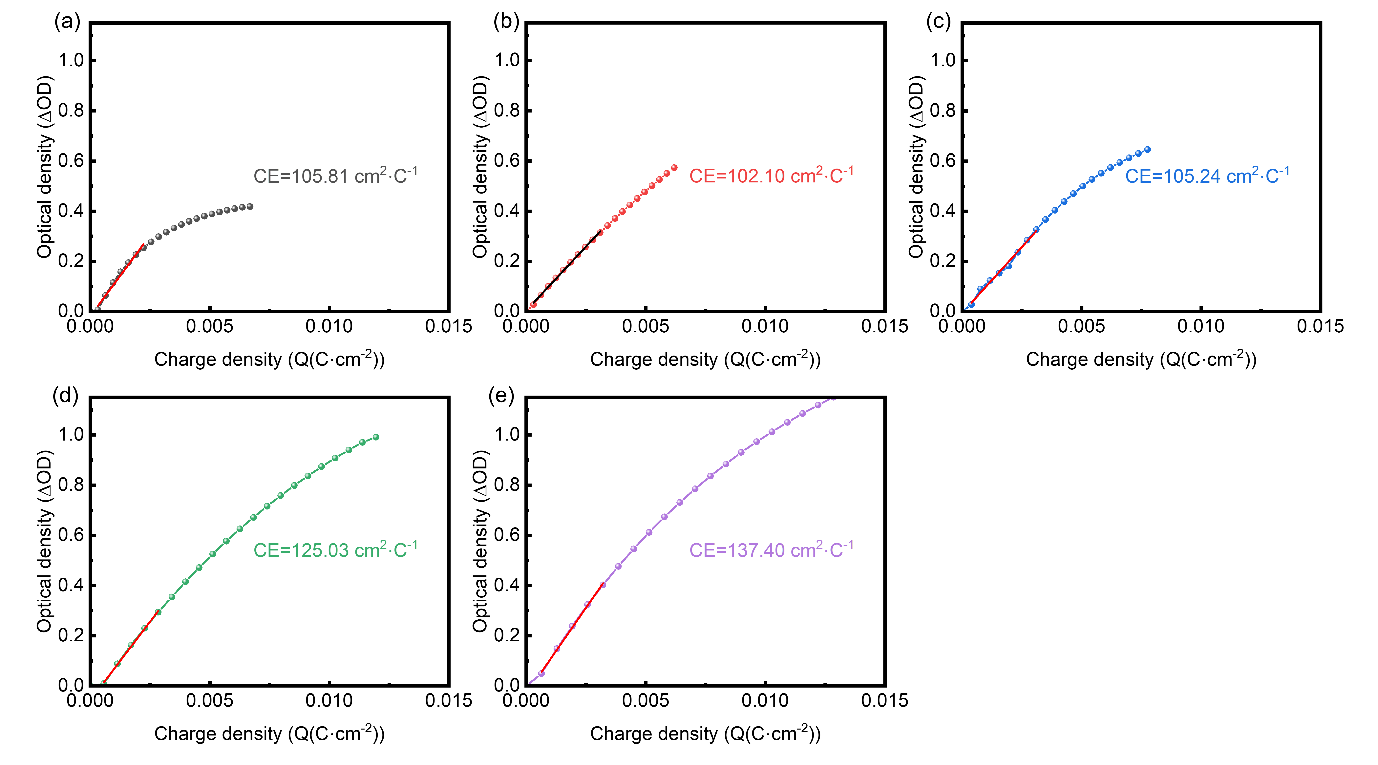
 Figure 4. CE fitting curves of different samples calculated from the optical density change versus charge density: (a) pure WO_3_, (b) 100 W TWO, (c) 150 W TWO, (d) 200 W TWO, (e) GTWO films.


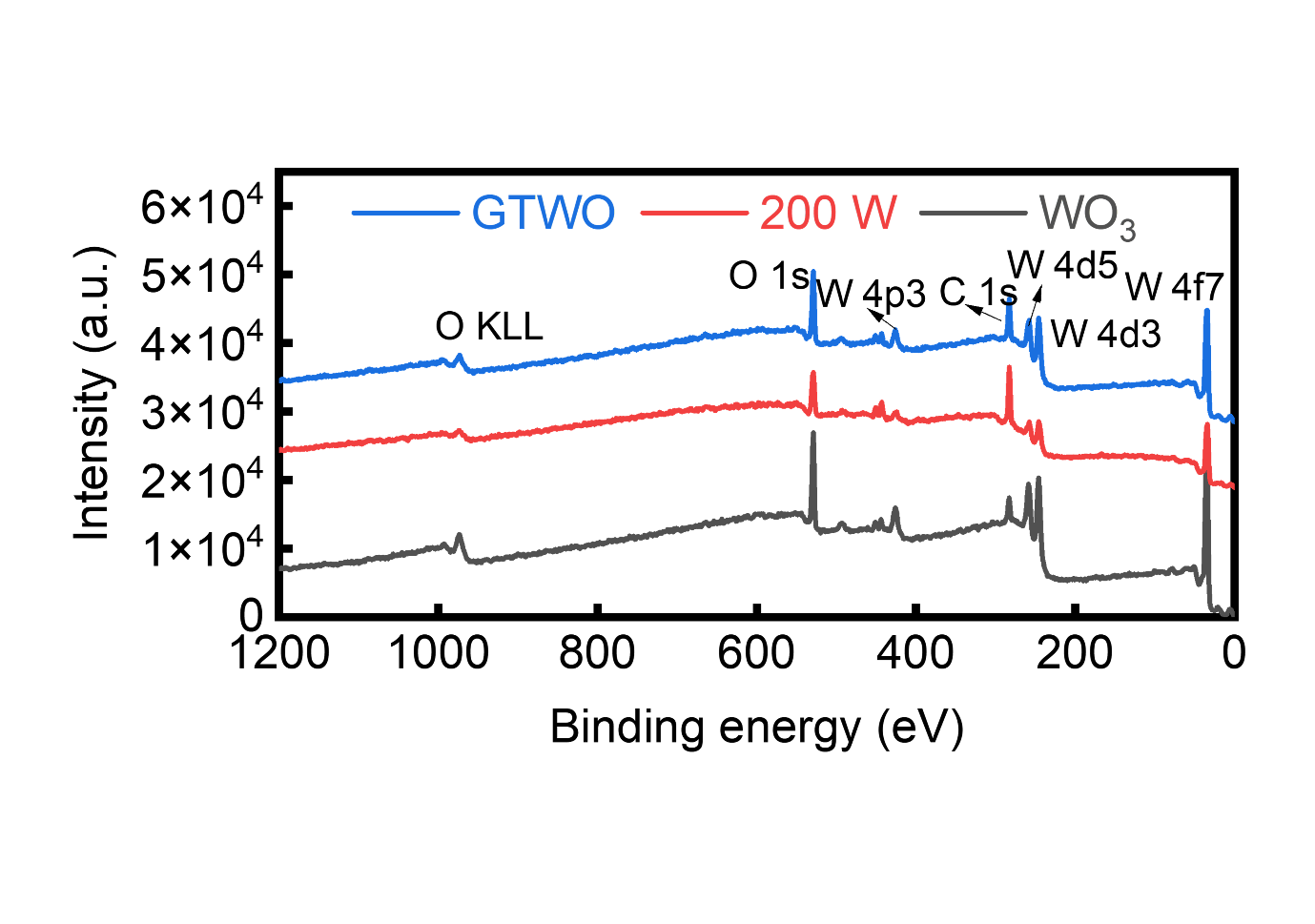


Figure 5. Surface XPS spectra comparison for pure WO_3_, uniformly doped TWO (200 W RF power of TiO_2_ target) and GTWO.


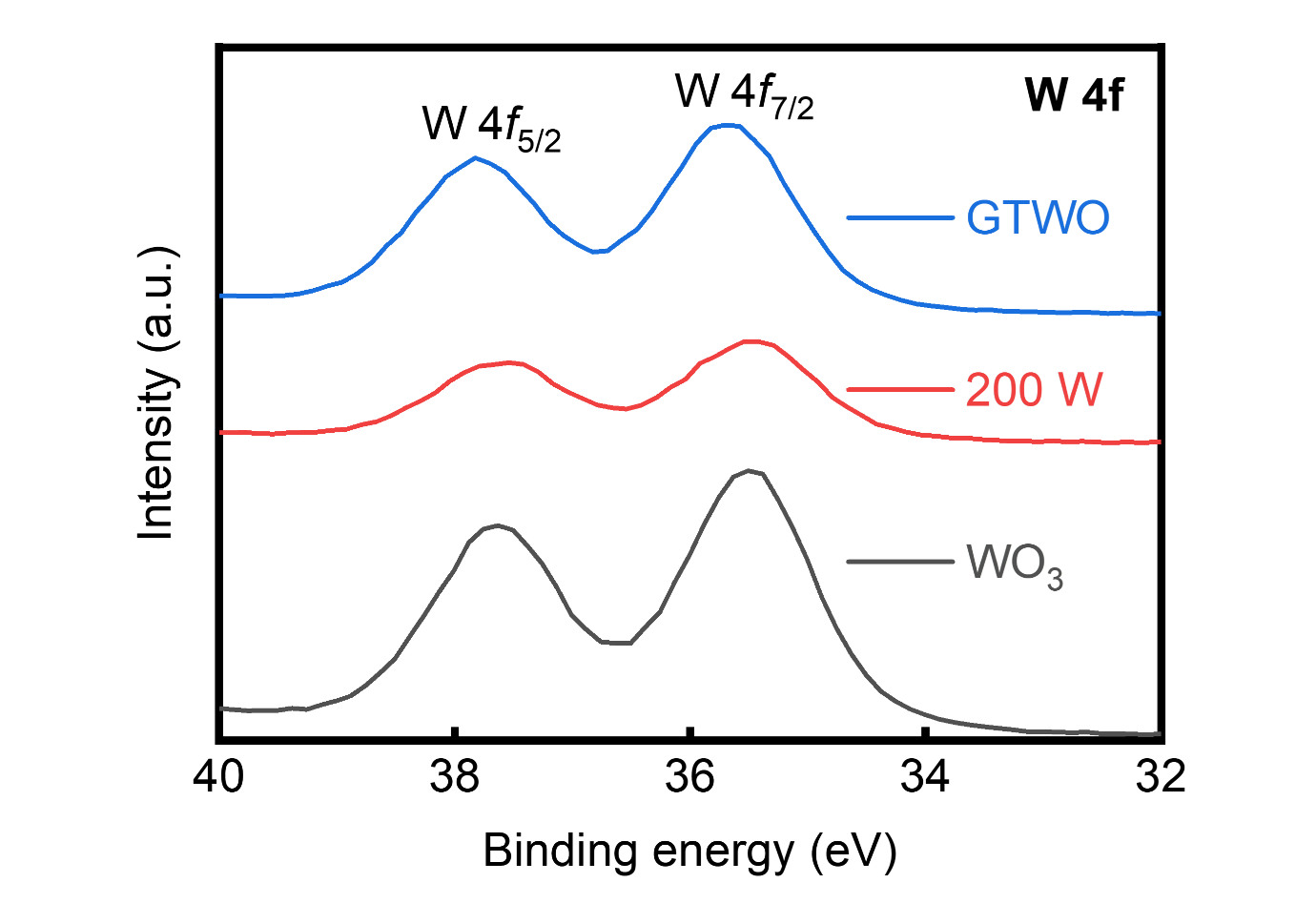


Figure 6. Surface W 4f spectra comparison for pure WO_3_, uniformly doped TWO (200 W RF power of TiO_2_ target) and GTWO.


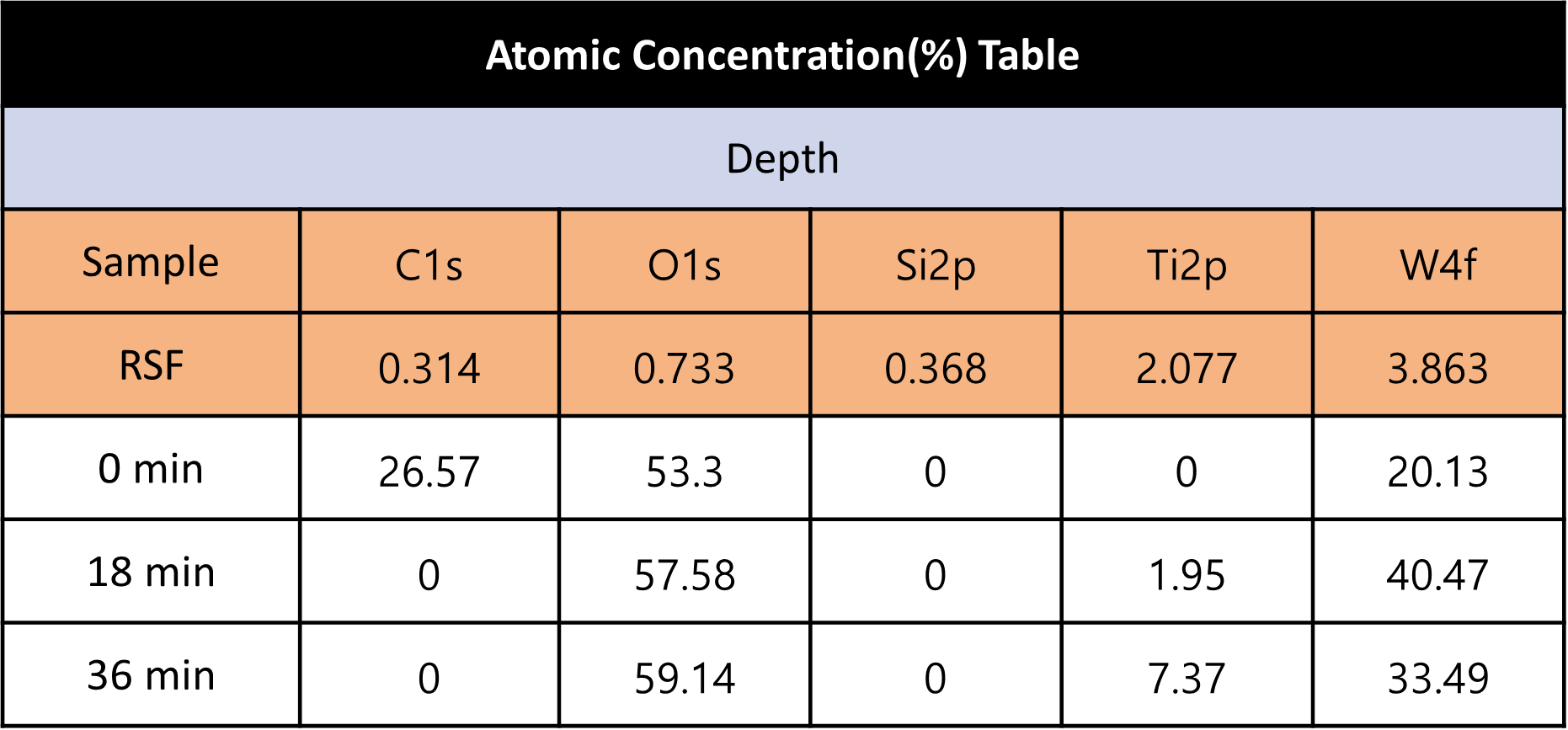


Figure 7. Atomic concentration of elements in the GTWO film at different sputtering depths determined by XPS depth profiling.


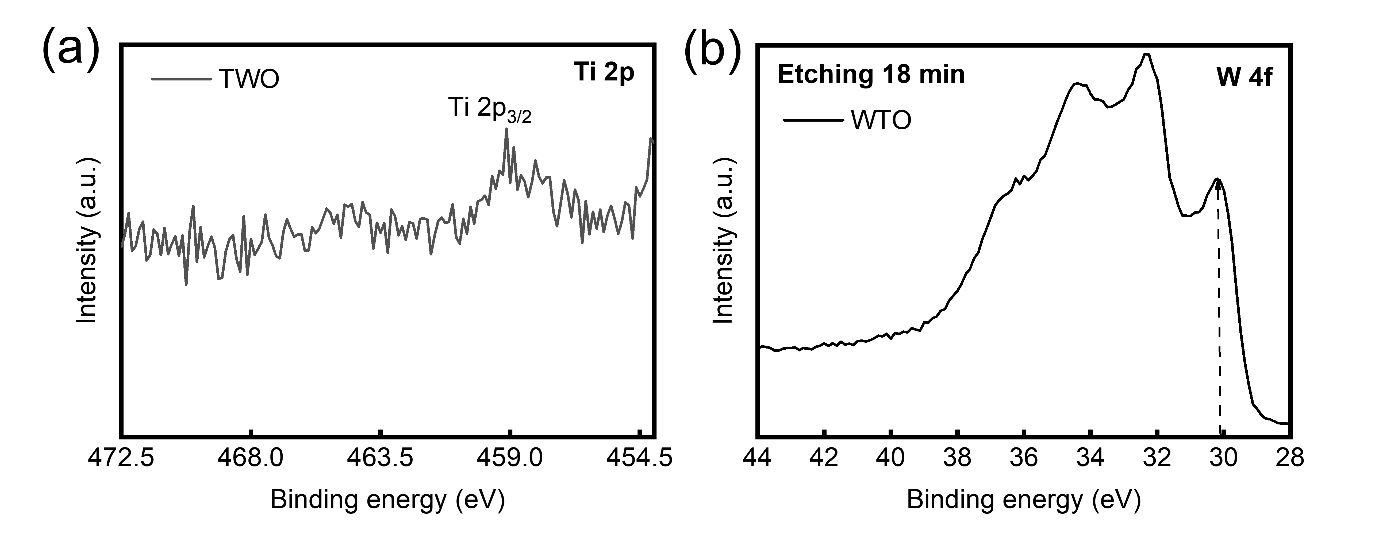


Figure 8. XPS spectra for TWO (200 W RF power of TiO_2_ target) after etching 18 min: (a) Ti 2p spectra, (b) W 4f spectra.


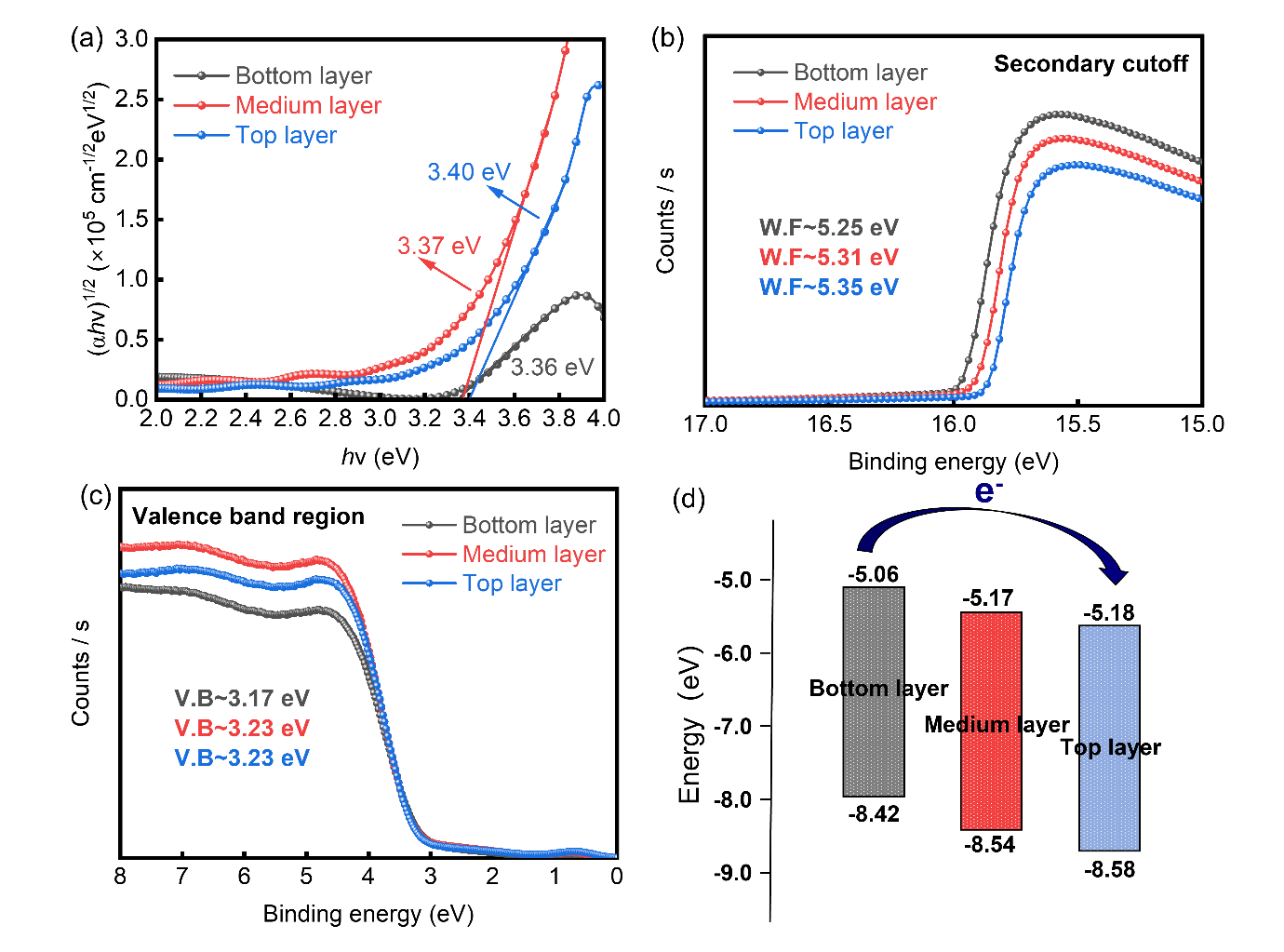


Figure 9. Comprehensive electronic structure characterization and energy band engineering of the GTWO film: (a) Tauc plots, (b) secondary cutoff, (c) valence band region, and (d) resulting energy band alignment scheme showing the built-in electric field.


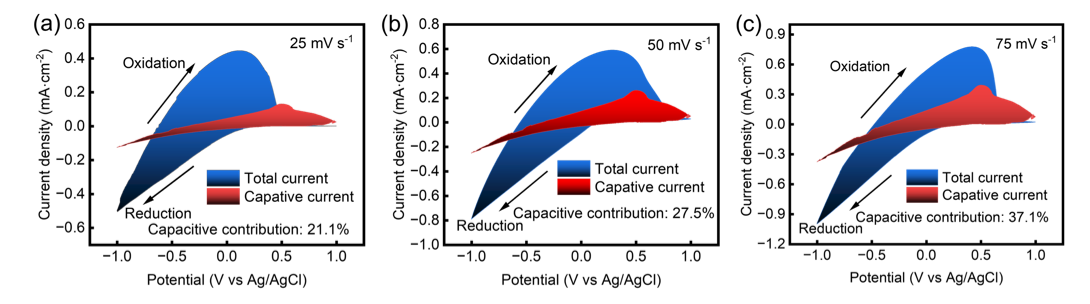


Figure 10. Schematic of capacitive contribution separation for GTWO under different scan rate: (a) 25 mV s^-1^, (b) 50 mV s^-1^ and (c) 75 mV s^-1^.


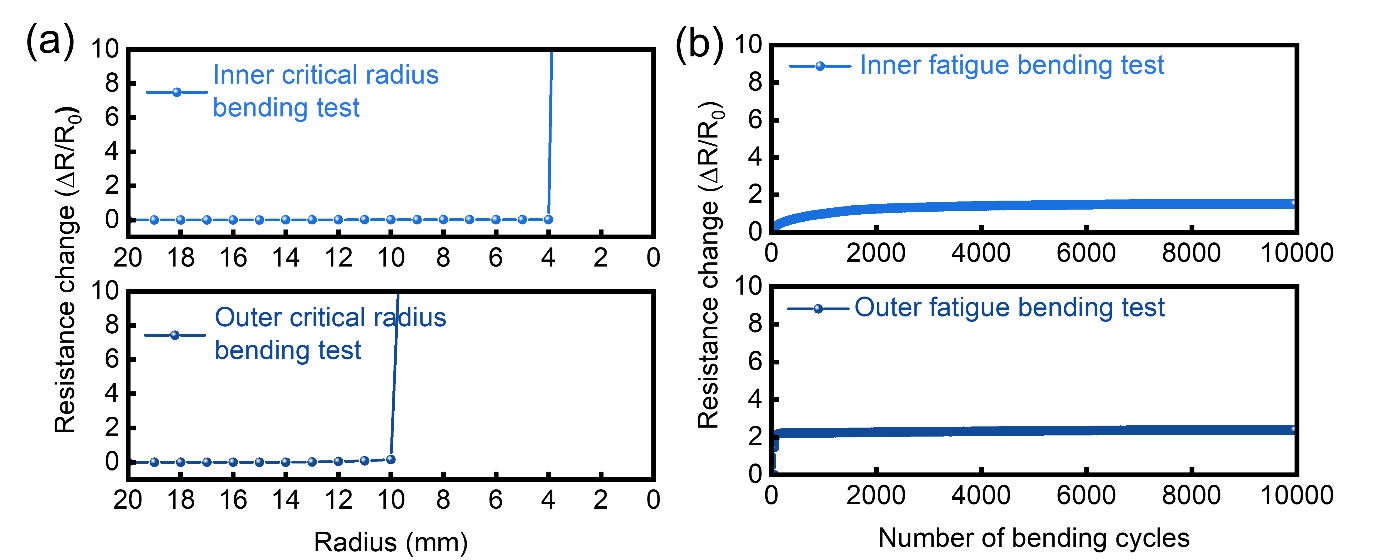


Figure 11. (a) Critical radius bending tests on inner and outer sides of TWO films. (b) Fatigue bending cycle tests (10,000 cycles) on inner and outer sides of TWO films.


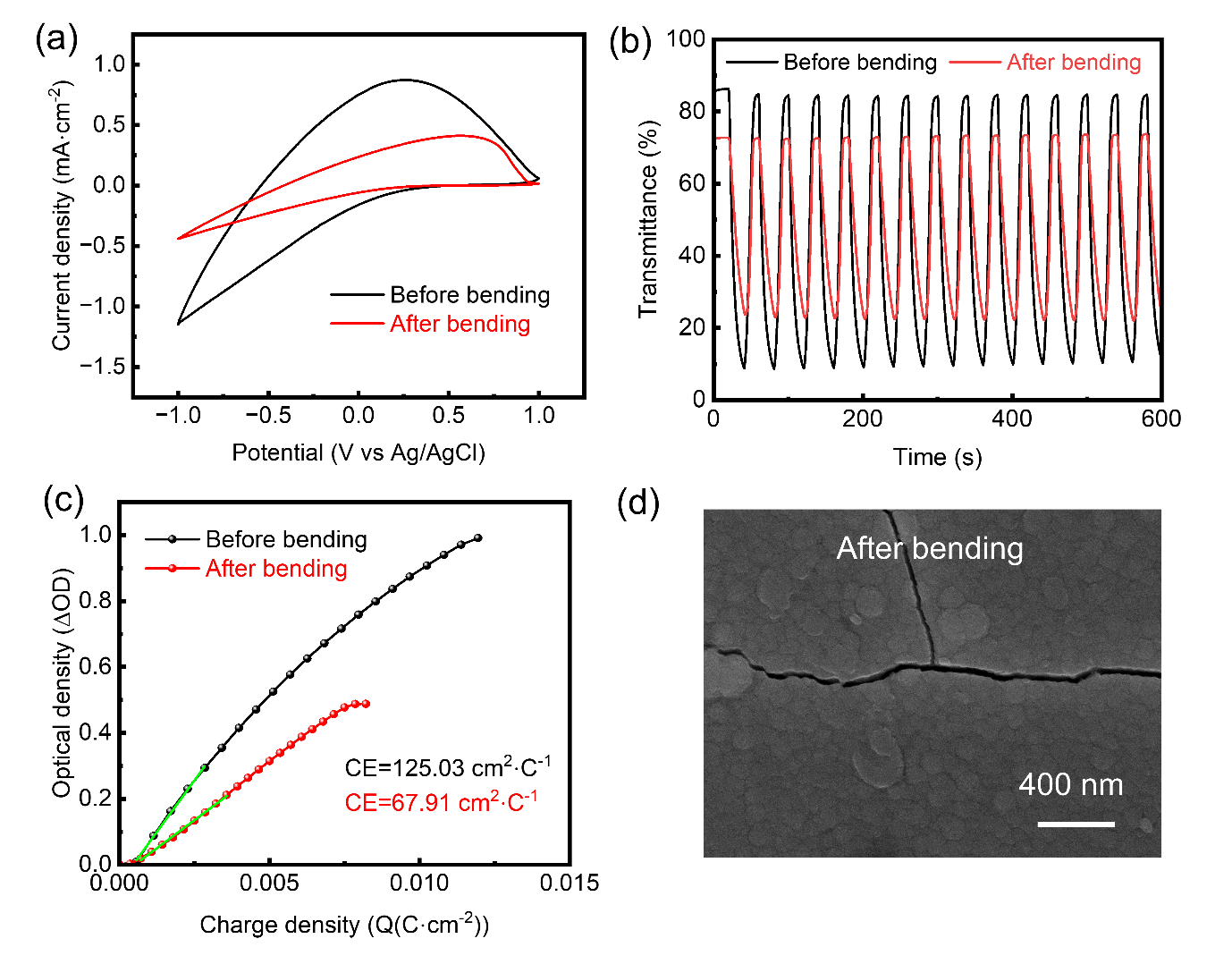


Figure 12. Comparison of electrical and optical properties of TWO films before and after bending: (a) CV curves before and after bending, (b) transmittance versus time curves at 630 nm before and after bending, (c) CE fitting curves calculated from the optical density change versus charge density, (d) FESEM image of surface morphology after bending.


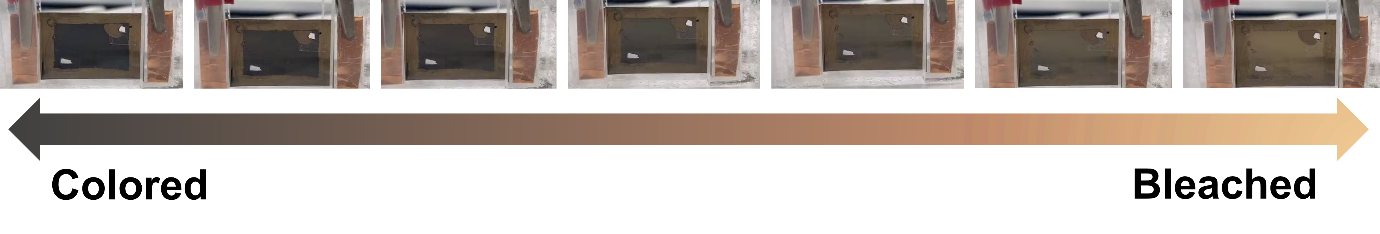


Figure 13. Images showing the ECD’s coloration from -2 V to bleaching at +2 V.


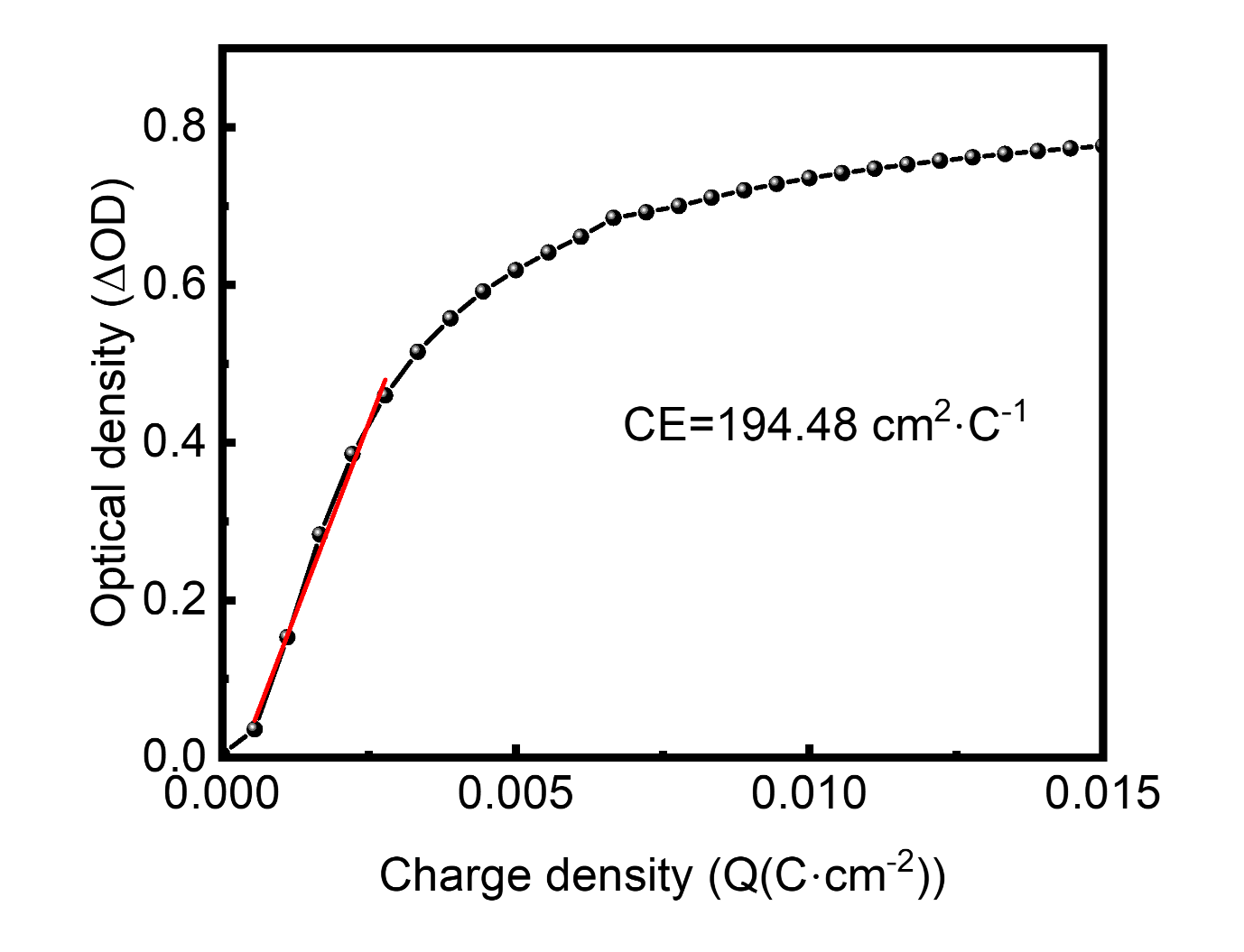


Figure 14. CE value of IGTO/GTWO/Electrolyte/ Mo–NiOx /IGTO ECD.

Table S1. Deposition parameters and thickness of TWO films at different TiO₂ RF powers

| RF powers of TiO_2_ [W] | Sputtering Times [s] | Thickness [nm] |
| --- | --- | --- |
| 0 | 1350 | 330 |
| 100 | 1325 | 332 |
| 150 | 1331 | 336 |
| 200 | 1337 | 335 |

*All films were deposited with comparable thickness (~330 nm) by adjusting the sputtering time accordingly.*
